# Morphological characteristics and transcriptome analysis at different anther development stages of the male sterile mutant MS7–2 in Wucai (*Brassica campestris* L.)

**DOI:** 10.1186/s12864-021-07985-5

**Published:** 2021-09-11

**Authors:** Jian Wang, Yitao Yang, Lei Zhang, Shaoxing Wang, Lingyun Yuan, Guohu Chen, Xiaoyan Tang, Jinfeng Hou, Shidong Zhu, Chenggang Wang

**Affiliations:** 1 College of Horticulture, Vegetable Genetics and Breeding Laboratory, Anhui Agricultural University, 130 West Changjiang Road, Hefei, 230036 Anhui China; 2 Provincial Engineering Laboratory for Horticultural Crop Breeding of Anhui, 130 West of Changjiang Road, Hefei, 230036 Anhui China; 3 Wanjiang Vegetable Industrial Technology Institute, Maanshan, 238200 Anhui China

**Keywords:** Wucai, Male sterility, Anther development, RNA-Seq, Differentially expressed genes

## Abstract

**Background:**

The discovery of male sterile materials is of great significance for the development of plant fertility research. Wucai (*Brassica campestris* L. ssp. *chinensis* var. *rosularis* Tsen) is a variety of non-heading Chinese cabbage. There are few studies on the male sterility of wucai, and the mechanism of male sterility is not clear. In this study, the male sterile mutant MS7–2 and the wild-type fertile plant MF7–2 were studied.

**Results:**

Phenotypic characteristics and cytological analysis showed that MS7–2 abortion occurred at the tetrad period. The content of related sugars in the flower buds of MS7–2 was significantly lower than that of MF7–2, and a large amount of reactive oxygen species (ROS) was accumulated. Through transcriptome sequencing of MS7–2 and MF7–2 flower buds at three different developmental stages (a–c), 2865, 3847, and 4981 differentially expressed genes were identified in MS7–2 at the flower bud development stage, stage c, and stage e, respectively, compared with MF7–2. Many of these genes were enriched in carbohydrate metabolism, phenylpropanoid metabolism, and oxidative phosphorylation, and most of them were down-regulated in MS7–2. The down-regulation of genes involved in carbohydrate and secondary metabolite synthesis as well as the accumulation of ROS in MS7–2 led to pollen abortion in MS7–2.

**Conclusions:**

This study helps elucidate the mechanism of anther abortion in wucai, providing a basis for further research on the molecular regulatory mechanisms of male sterility and the screening and cloning of key genes in wucai.

**Supplementary Information:**

The online version contains supplementary material available at 10.1186/s12864-021-07985-5.

## Background

Wucai (*Brassica campestris* L. ssp. *chinensis* var. *rosularis* Tsen) is a variant of non-heading Chinese cabbage [1, 2]. As an important autumn and winter vegetable, wucai is widely planted in the Yangtze-Huai River Basin. Due to its high nutritional value, wucai is becoming increasingly popular all over the world.

Male sterility in *Brassica* crops is considered as an ideal pollination control system for hybrid seed production. Wucai is one of the crops with substantial heterosis. Therefore, male sterile lines have long been used to produce hybrids that utilize heterosis effectively [3]. Based on the inheritance or origin, male sterility is classified into cytoplasmic male sterility (CMS), genetic male sterility (GMS), and cytoplasmic-genetic male sterility (CGMS) [4]. Two types of male sterility occur in wucai, including CMS and GMS. The sterility of CMS is controlled by the cytoplasmic genome, which is maternally inherited, and its fertility can be restored by restoring genes located in the nuclear genome [5]. In addition, CMS has some issues, such as incomplete fertility and low combining ability [6]. GMS can theoretically overcome the drawbacks of CMS. Compared with CMS, GMS has an obvious disadvantage, that only 50% of maternal plants are male sterile plants, which requires the removal of the remaining 50% of male fertile plants [7, 8]. However, GMS is becoming more and more popular in commercial seed production because of its advantages of relatively complete fertility, no effect of cytoplasmic type, wide restorer lines, and highly pure hybrid F1 seeds [9].

Stamen development plays an important role in the sexual reproduction of flowering plants, whereas its development process is very complex. Anthers are important organs in sexual plant reproduction. Any event or gene abnormality in anther development may affect male fertility [10]. The mature anther is surrounded by four layers of cells, including the epidermis, which provides environmental protection for the interior of the pollen grains [11]; the endothecium and middle layer, which transport nutrients; and the tapetum, which transports nutrients to the pollen sac [12].

In the early stage of anther development, primary peripheral cells differentiate into primary parietal cells and primary sporophytic cells, and primary peripheral cells differentiate into the endothecium (fibrous layer), the middle layer, and the tapetum. In addition, callose begins to deposit in the plasma membrane and primary wall until surrounding the entire mother cell. In the tetrad stage, the tapetum reaches the maximum and most active stage and begins to secrete callase. The callose gradually degrades, and the microspores become separated. The pollen grains gradually form in the pollen sac, and the anther volume expands gradually, and at this stage, the tapetum undergoes programmed cell death (PCD). In the late stage of anther development, mature pollen grains are released from the pollen sac, and the anther cells tend to senesce, resulting in anther abscission [13]. The production of viable pollen requires the PCD of the tapetum at an appropriate time, which is strictly regulated by many genes [14]. In previous studies, reactive oxygen species (ROS) have been involved in the process of PCD [15, 16]. The metabolism of ROS in plants is disturbed during PCD, which leads to abnormal tapetum degradation, resulting in male sterility [17–19].

Thus far, at least a hundred GMS genes have been identified in plants [20–22]. *Male Sterile2* (*MS2*) is predicted to encode a fatty acid reductase required for pollen wall development in *Arabidopsis* [23]. *CYP704B1* catalyzes the co-hydroxylation of long-chain fatty acids and participates in the synthesis of sporopollenin [24]. *UDT1* and *PTC1* play an important role in tapetum development [25, 26]. In rice, the tapetum of the *udt1* mutant failed to differentiate and vacuolize in the early stage of meiosis, the degeneration of the intermediate layer was inhibited, and microspore development failed, leading to no pollen production in the anther locules. *PTC1* encodes a PHD-finger (for plant homeodomain) protein. The *ptc1* mutant exhibited an abnormal proliferation of the tapetum during microspore development and delayed degradation and abnormal pollen wall formation, leading to microspore development suspension. If the molecular mechanism of genic male sterility can be elucidated, it will be possible to manipulate male sterility via genetic engineering [27]. Many transcription factors have been found to regulate male sterility, mainly by affecting the development of the tapetum and pollen wall. *DYT1* encodes a putative bHLH transcription factor that affects the expression of many tapetum-related genes, which is a crucial part of the genetic network controlling anther development [28]. *TDF1* encodes a putative R2R3 MYB transcription factor and regulates the differentiation of the tapetum, which acts downstream of *DYT1* and is essential for early tapetal development [29, 30]. *AMS* is a typical transcription factor belonging to the MYC sub-family of bHLH genes that plays an important role in tapetal cell development and post-meiotic microspore transcription regulation during anther development [31]. The genetic pathway of *DYT1*-*TDF1*-*AMS* has been proven to regulate the late development of the tapetum [32, 33].

RNA sequencing (RNA-Seq) technology is a high-throughput transcriptome analysis platform that can comprehensively and accurately detect gene expression patterns. It has been applied to many horticultural crops, including Chinese cabbage [34, 35], celery [36], pepper [37], and wucai [38]. This study aims to further elucidate the differences in the physiology and transcriptome between the male sterile mutant MS7–2 and wild-type fertile plant MF7–2. According to the abortion period of MS7–2, we isolated mRNA from the different developmental stages of MS7–2 and MF7–2 flower buds and performed genome-wide transcription profiling. Physiological assessment showed that sugar synthesis in the MS7–2 flower buds was inhibited, and a large amount of ROS was accumulated. The results of this study help further elucidate the molecular mechanisms of male sterility of wucai, providing useful information for the further development of heterosis breeding.

## Results

### Phenotypic and cytological characterization of MS7–2

**Fig. 1:**
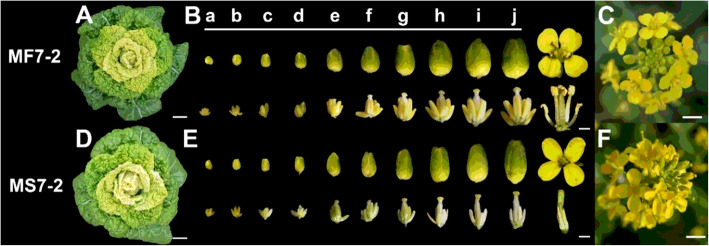
Phenotypic characterization of MF7–2 and MS7–2 floral buds. (**A**–**C**) Phenotype of MF7–2 and (**D**–**F**) phenotype of MS7–2. (**A** and **D**) Individual plants, bar = 2 cm; (**B** and **E**) floral buds during the young stage to anthesis (stages a–j) of MF7–2 and MS7–2, bar = 1 mm; (**C** and **F**) inflorescence, bar = 2 cm

The phenotypic characterization comparison of the MS7–2 and MF7–2 lines showed that MS7–2 exhibited no significant differences in morphological indicators such as plant type and leaf color from MF7–2 **(**Fig. 1 A, D). The main difference was in the floral organs. The anthers of MF7–2 developed normally and possessed a large number of plump pollen grains, while the filaments of MS7–2 were short and the anthers were abnormally degraded, with no obvious pollen grains on the surface **(**Fig. 1 B, C, E and F). According to the stage of anther development, we divided the flower buds into 10 levels (a–j). At the a and b stages, there was no significant difference between the fertile and sterile anthers, but with the development of the buds, the anthers of MF7–2 gradually matured and became full, while the anthers of MS7–2 became short and shrunken. When the anthers of MF7–2 had fully flowered, a large number of pollen grains were produced, and the filaments were elongated, while the filaments of MS7–2 were very short, and the anthers were completely shrunken and could not produce pollen grains **(**Fig. 1 B, C).

**Fig. 2:**
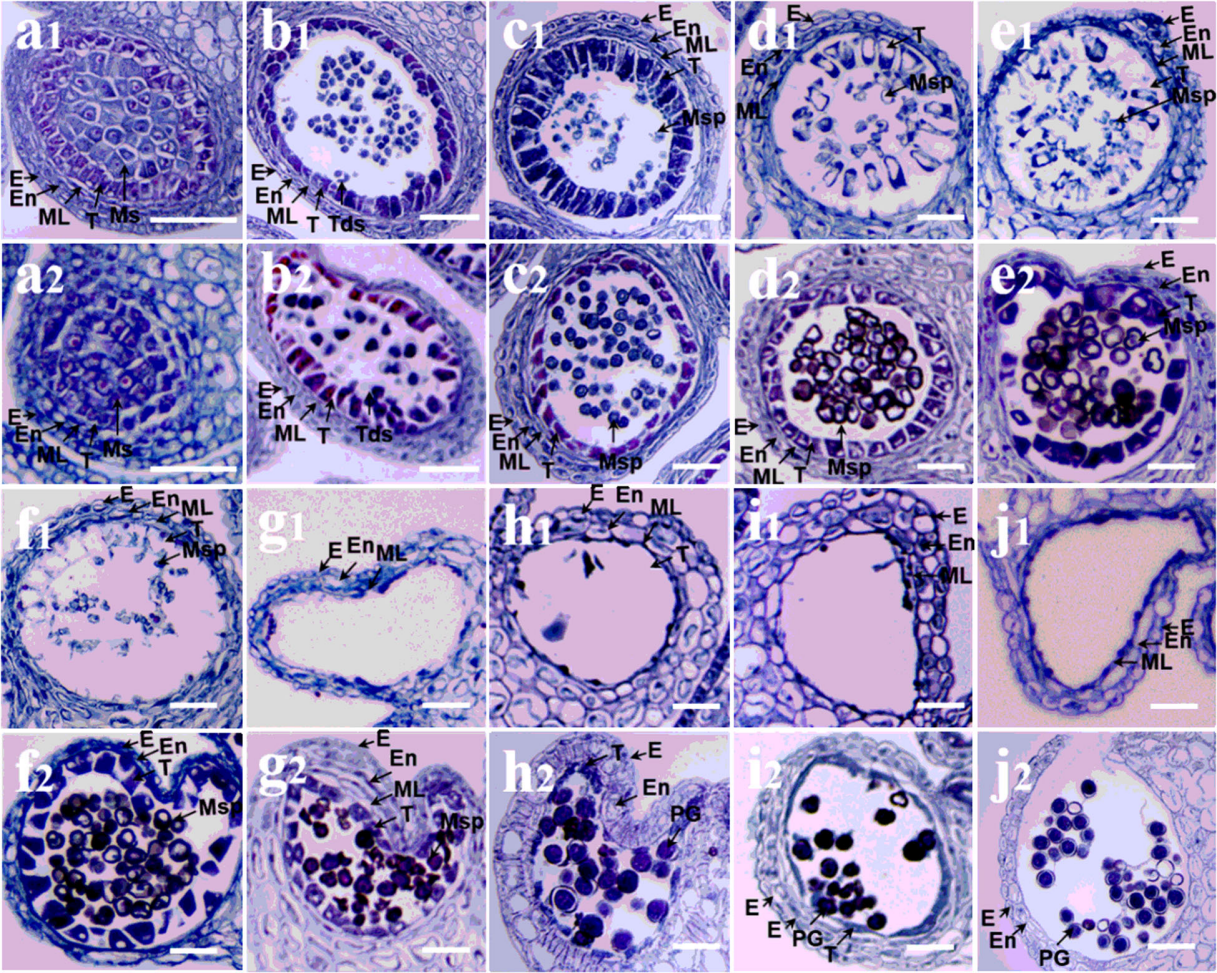
Paraffin sections of the corresponding size of MS7–2 anthers (a1–j1) and MF7–2 anthers (a2–j2) of wucai. (a1–j1) MS7–2 (< 1.0 mm; 1.0–1.5 mm; 1.5–2.0 mm; 2.0–2.25 mm; 2.25–2.5 mm; 2.5–2.75 mm; 2.75–3.0 mm; 3–3.5 mm; 3.5–4.0 mm; 4.0–4.5 mm), bar =2 mm. (a2–j2) MF7–2 (< 1.0 mm; 1.0–1.5 mm; 1.5–2.0 mm; 2.0–2.25 mm; 2.25–2.5 mm; 2.5–2.75 mm; 2.75–3.0 mm; 3–3.5 mm; 3.5–4.0 mm; 4.0–4.5 mm), bar =2 mm. E, epidermis; En, endothecium; ML, middle layer; Ms., meiocytes; Msp, microspores; PG, pollen grain; Sp, sporogenous cell; T, tapetum; Tds, tetrads

In order to accurately identify the cause of the pollen abortion, we observed paraffin sections of the two lines (Fig. 2). The results of the paraffin sectioning showed that there was no significant difference in anther development between the pollen mother cell stage and tetrad stage (Fig. 2 a1, b1, a2, and b2). However, after the tetrad stage, the tapetum of MS7–2 developed abnormally and began to expand. Microspores accumulated in the anther chamber (Fig. 2 c1–f1), and the mononuclear microspore of MF7–2 formed mature pollen grains through mitosis (Fig. 2 c2–j2). However, in MS7–2, the tapetum cells continued to expand, and the microspores were squeezed, and thus there was not enough space for their development into pollen grains, which led to abortion (Fig. 2 g1–j1). At the late stage of anther development, the tapetum of MF7–2 gradually degenerated, the cells in the middle layer gradually thinned and finally degenerated, and the mature pollen grains were released after formation (Fig. 2 g2–j2).

### Measurements of physiological indices in MS7–2

**Fig. 3:**
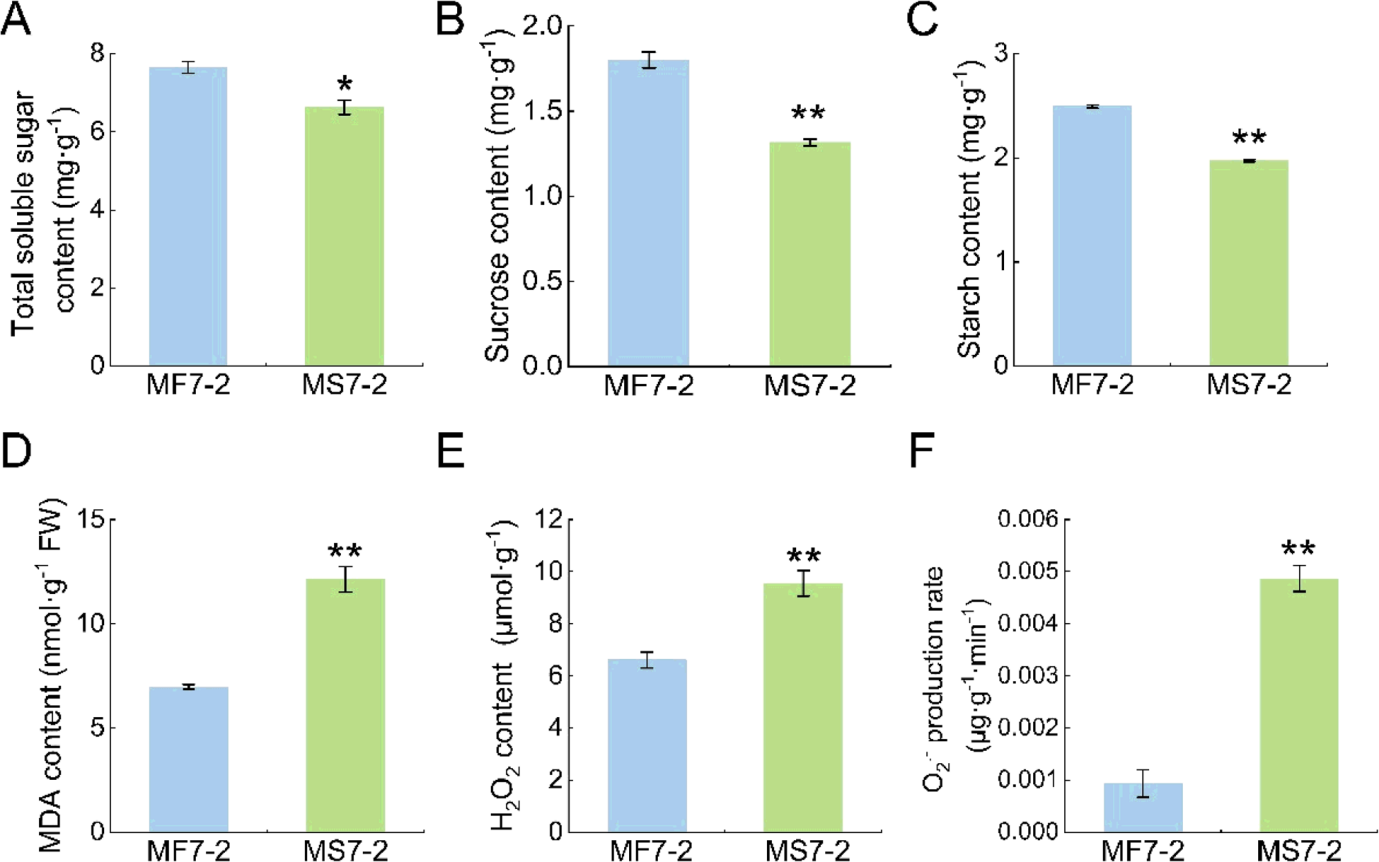
Determination of the physiological indices of MS7–2 and MF7–2. (**A**) Determination of soluble sugar content. (**B**) Determination of sucrose content. (**C**) Determination of starch content. (**D**) Determination of MDA content. (**E**) Determination of H_2_O_2_ content. (**F**) Determination of O_2_^.-^ production rate

Physiological indices such as energy metabolism and antioxidant metabolism were measured in the buds of MS7–2 and MF7–2. Carbohydrates provide energy for anther development and synthesis, and excessive accumulation of ROS in cells can damage the cell membrane system, resulting in intracellular metabolic disorder. The results showed that the contents of soluble sugar, sucrose, and starch in the buds of MS7–2 were significantly lower than those of MF7–2, indicating that the substance metabolism in the buds of MS7–2 was affected, and the synthetic transportation of sugars was inhibited, which may have led to male sterility **(**Fig. 3 a, b, and c). In addition, in terms of antioxidant metabolism, the MDA and H_2_O_2_ levels in the buds of MS7–2 were significantly increased compared with MF7–2, and the superoxide anion production rate was also significantly higher than in MF7–2 **(**Fig. 3 d, e, and f). This result indicated that ROS accumulated in MS7–2, and damage to the membrane was incurred.

### Transcriptome analysis

**Fig. 4:**
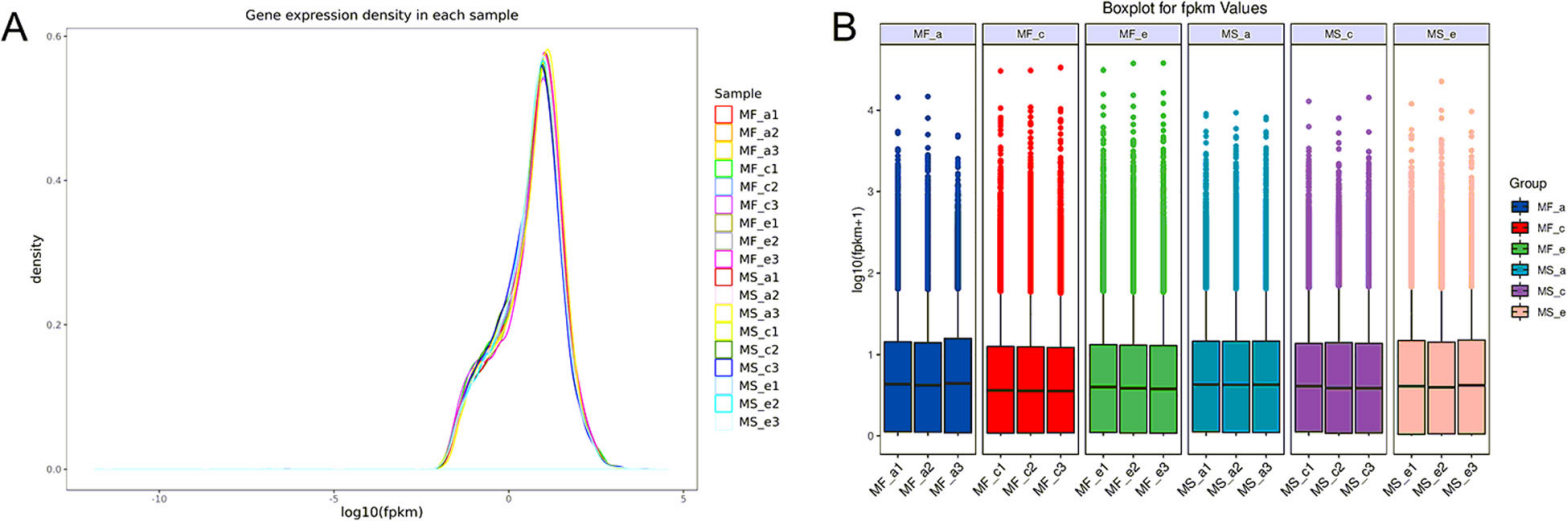
FPKM density distribution curve and box plot of each sample. (**A**) The curves of different colors in the figure represent different samples, the abscissa of the points on the curve represents the logarithm of the corresponding sample FPKM, and the ordinate of the points represents the probability density. (**B**) The abscissa is the sample name, the ordinate is log10 (FPKM+ 1), and the box graph pairs of each region have five statistics (the maximum, upper quartile, median, lower quartile, and minimum from top to bottom)

In order to explore the molecular mechanism underlying the microspore abortion observed in MS7–2, we selected the three developmental stages mentioned above, i.e., stage a (microspore mother cell stage) with no significant difference, stage c (mononuclear microspore stage) when changes begin, and stage e (late mononuclear microspore stage) with great differences, for transcriptome analysis. Raw reads were filtered to remove low-quality reads, and a total of 846,213,182 clean reads were ultimately used in the alignment. The percentage of sequences with nucleotide mass fraction Q30 values greater than 30 was 94.61% in all samples, and the GC content was 46.01% **(**Table 1**)**. By comparing the reads to the reference genome, the genome alignment of each sample was obtained, and the comparison rate was 88.85–89.86%. After directly comparing the density and discrete distributions of the expression levels for different samples, we found that the sequencing quality and gene expression levels were basically the same **(**Fig. 4 a, b). Thus, the results indicated that the quality of the data obtained by sequencing was sufficient, and that the data could be analyzed in the next step.

**Table 1:** Illumina sequencing data and results of de novo assembly

Sample	Total reads	Q30	GC content	Total mapped	Multiple mapped	Uniquely mapped
MF_a1	47,261,182	94.58%	46.16%	42,249,106(89.39%)	1,153,287(2.44%)	41,095,819(86.95%)
MF_a2	39,720,798	94.66%	45.89%	35,440,440(89.22%)	958,474(2.41%)	34,481,966(86.81%)
MF_a3	39,605,112	94.49%	46.22%	35,190,113(88.85%)	978,840(2.47%)	34,211,273(86.38%)
MF_c1	52,506,816	94.85%	45.93%	47,180,496(89.86%)	1,282,201(2.44%)	45,898,295(87.41%)
MF_c2	43,897,316	94.49%	45.89%	39,425,100(89.81%)	1,041,897(2.37%)	38,383,203(87.44%)
MF_c3	47,741,050	94.51%	45.56%	42,769,446(89.59%)	1,103,699(2.31%)	41,665,747(87.27%)
MF_e1	51,530,136	94.55%	45.43%	46,108,970(89.48%)	1,219,597(2.37%)	44,889,373(87.11%)
MF_e2	48,881,998	94.42%	45.39%	43,747,842(89.50%)	1,189,445(2.43%)	42,558,397(87.06%)
MF_e3	49,059,912	94.56%	45.46%	43,936,829(89.56%)	1,146,466(2.34%)	42,790,363(87.22%)
MS_a1	46,995,876	94.69%	46.28%	41,910,271(89.18%)	1,180,814(2.51%)	40,729,457(86.67%)
MS_a2	50,035,570	94.63%	46.47%	44,747,784(89.43%)	1,221,996(2.44%)	43,525,788(86.99%)
MS_a3	40,922,432	94.77%	46.46%	36,570,701(89.37%)	998,232(2.44%)	35,572,469(86.93%)
MS_c1	45,571,650	94.51%	46.30%	40,751,380(89.42%)	1,149,356(2.52%)	39,602,024(86.90%)
MS_c2	50,301,580	95.16%	46.44%	45,059,996(89.58%)	1,253,441(2.49%)	43,806,555(87.09%)
MS_c3	48,264,128	94.40%	46.16%	43,156,288(89.42%)	1,224,968(2.54%)	41,931,320(86.88%)
MS_e1	46,593,432	94.54%	45.98%	41,571,814(89.22%)	1,047,534(2.25%)	40,524,280(86.97%)
MS_e2	49,135,418	94.52%	45.94%	43,920,962(89.39%)	1,110,749(2.26%)	42,810,213(87.13%)
MS_e3	48,188,776	94.63%	46.21%	42,986,555(89.20%)	1,141,293(2.37%)	41,845,262(86.84%)
Total	846,213,182	94.61%	46.01%	756,724,093(89.42%)	20,402,289(2.41%)	736,321,804(87.00%)

### Identification of DEGs

**Fig. 5:**
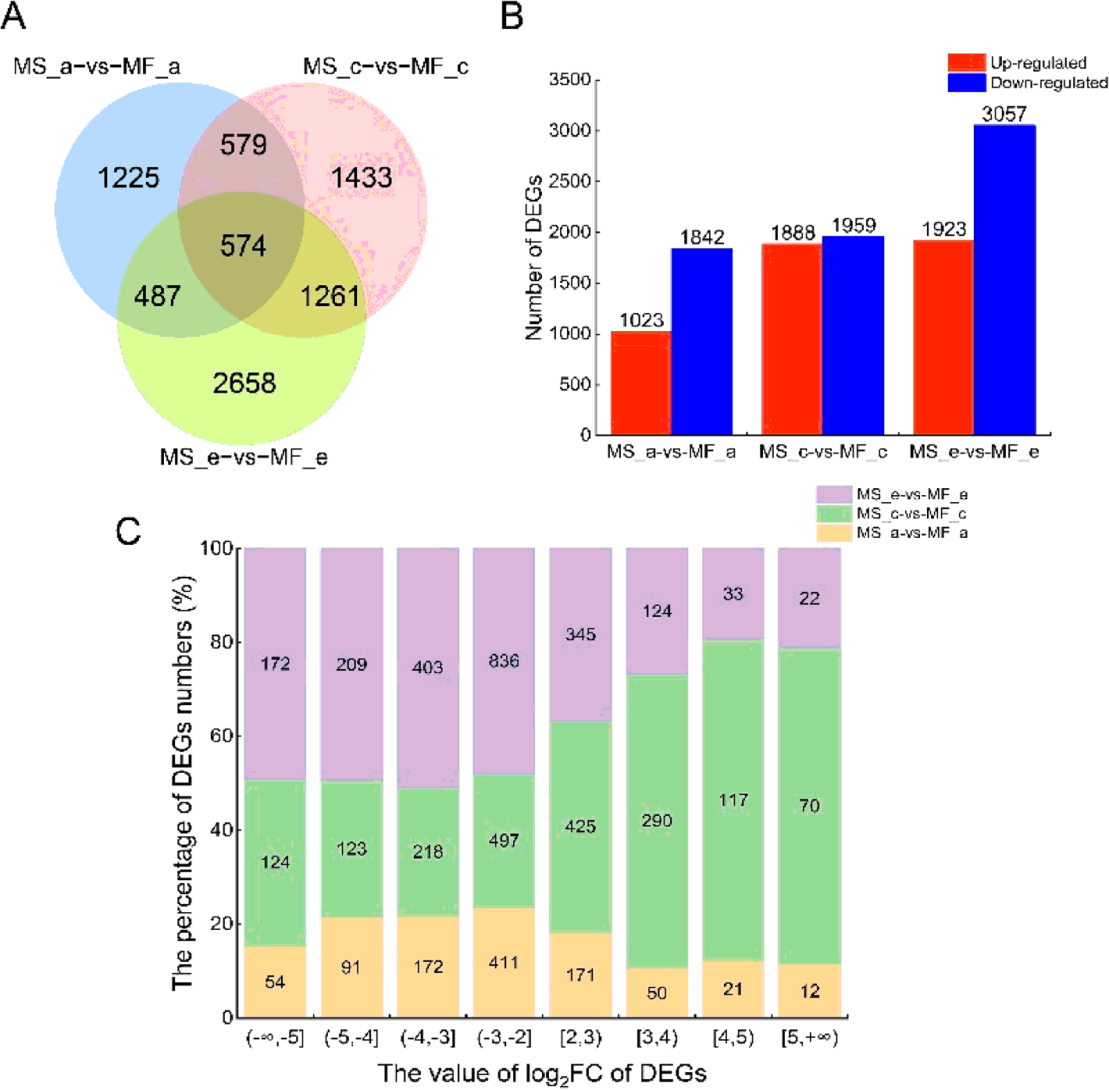
Analysis of DEGs in MS7–2 compared to MF7–2 from the three stages of anther development. (**A**) Venn diagrams showing the DEGs shared among the three stages. (**B**) Number of DEGs that were up- or down-regulated in the three development stages. (**C**) The distribution of the log2Ratio of DEGs in MS7–2 compared to MF7–2

The DEGs were identified using the DESeq (2012) R package functions estimate Size Factors and nbinom Test. The threshold for DEG identification was a *P*-value< 0.05 and FC > 2 or FC < 0.5. In MS7–2 and MF7–2, a total of 8217 DEGs were identified, of which 574 DEGs (390 down-regulated and 184 up-regulated) were shared among the three groups **(**Fig. 5 a). Notably, with the development of the anthers, the number of DEGs showed an increasing trend. In the c stage when the differences began, the number of up-regulated and down-regulated genes was basically the same, and 4981 DEGs were identified in the e stage when the differences were relatively large. Additionally, there were more down-regulated genes than up-regulated genes **(**Fig. 5 b). Of the up-regulated genes, 1426 DEGs exhibited more than two-fold change in gene expression in the c and e stages, whereas only 254 DEGs in the a stage exhibited more than two-fold change. Among the down-regulated genes, 2582 DEGs exhibited over two-fold expression differences in the c and e stages, whereas only 728 DEGs demonstrated this in the a stage **(**Fig. 5 c). These results may indicate that in the c and e stages of anther development, the DEGs may contain more key information related to male sterility.

### GO and pathway analysis of DEGs

**Fig. 6:**
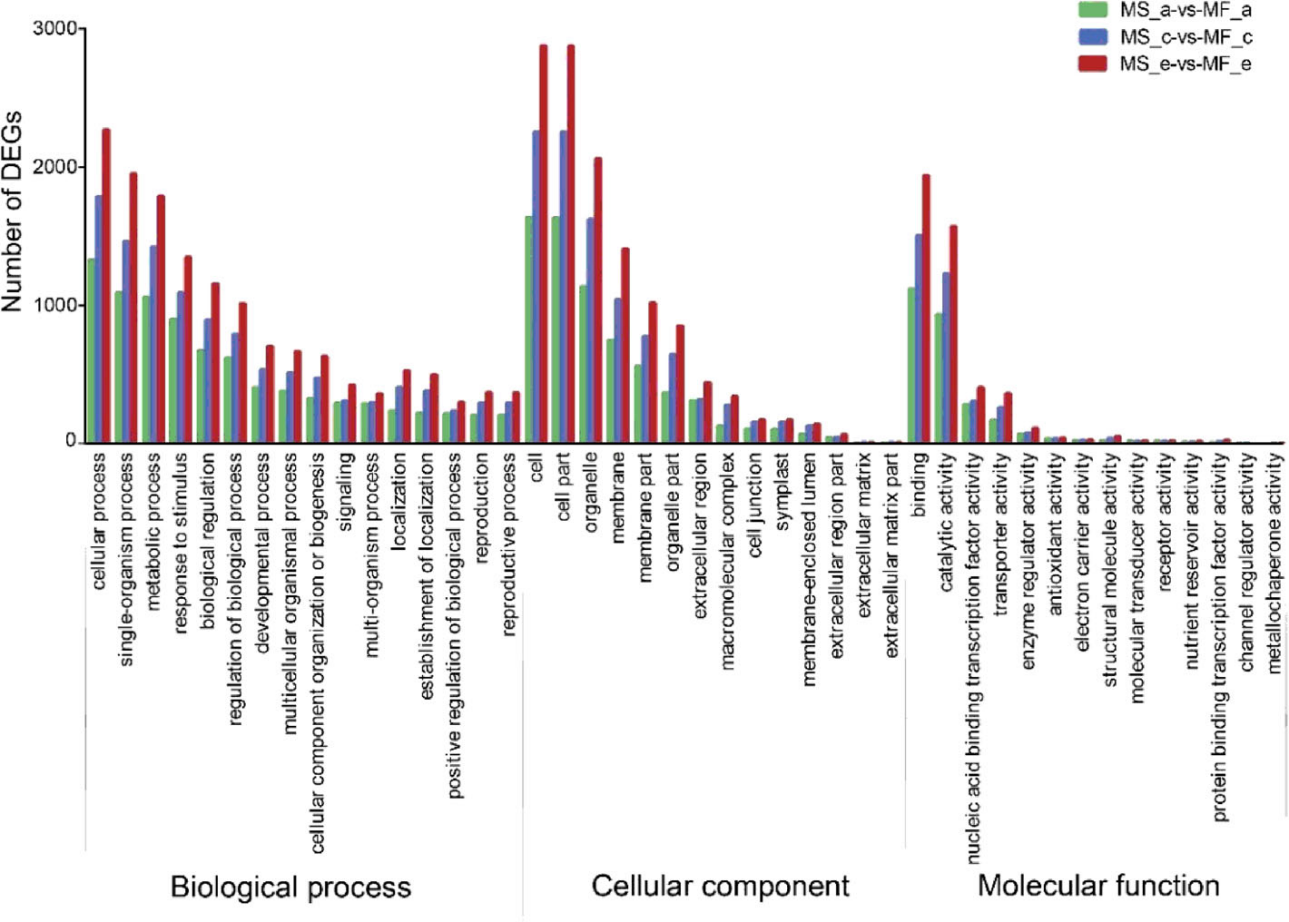
GO functional enrichment of DEGs between MS7–2 and MF7–2 from three stages of anther development

GO enrichment analysis was used for the global analysis of DEGs. The DEGs were classified into three categories of ontologies including biological process, molecular function, and cellular component. The DEGs of MS and MF were mainly distributed in 53 GO terms. In the biological process category, the DEGs in the three stages were mainly involved in cellular process, metabolic process, and single-organism process. In the cellular component category, cell, cell part, and organelle were highly enriched in all stages. In the molecular function category, the DEGs enriched in binding and catalytic activity were the most abundant **(**Fig. 6**)**. As also indicated in Fig. 6, the number of DEGs enriched in the c and e stages was higher than that in the a stage. These results indicated that multiple complex metabolic pathways were involved in anther development in MS, and anther development in MS and MF was likely to be different from the tetrad period, which is consistent with our cytological observations.

KEGG pathway enrichment was used to identify the major biochemical and signal transduction pathways that the DEGs participated in. A total of 639, 988, and 1212 DEGs in the three stages were significantly annotated in KEGG pathways, respectively. These DEGs were enriched in 122 KEGG pathways.

**Fig. 7:**
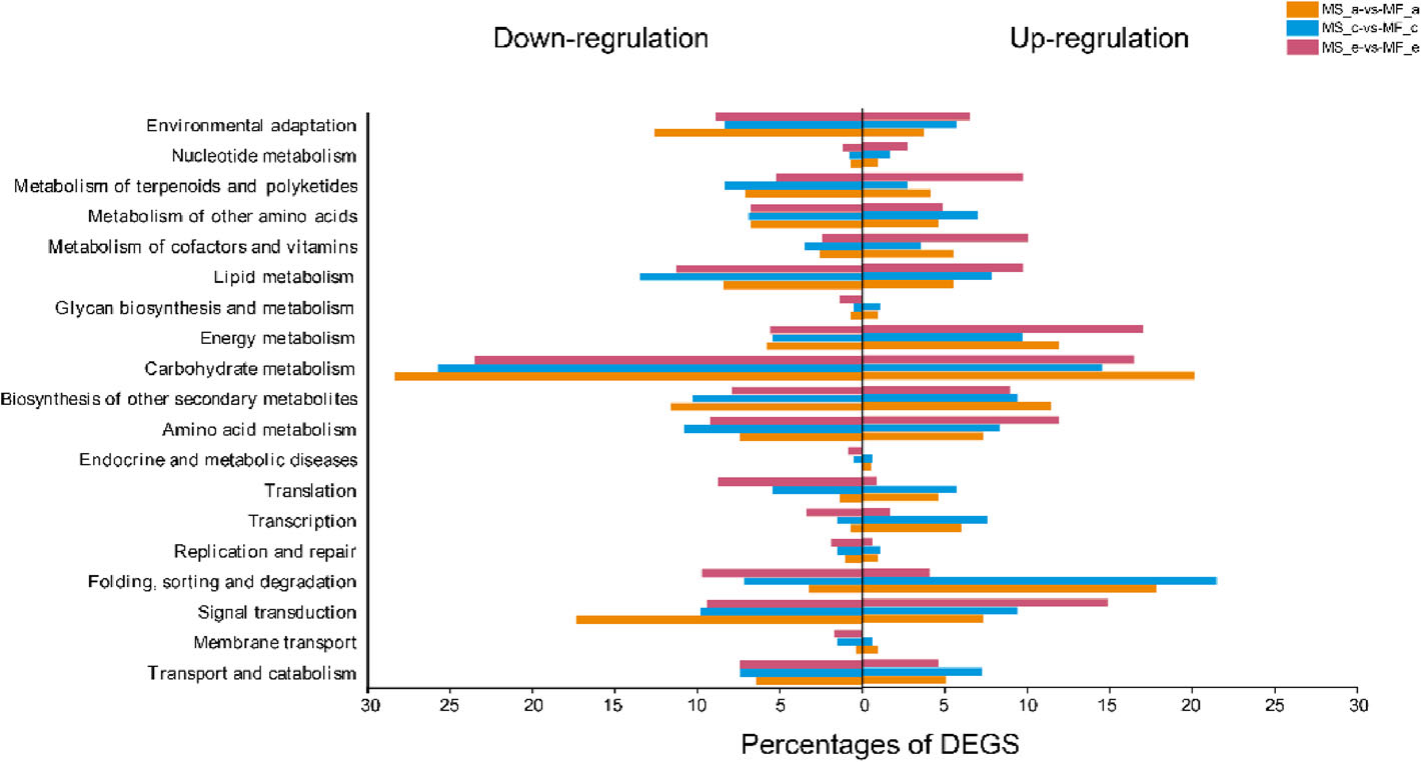
KEGG enrichment of DEGs between MS7–2 and MF7–2 from three stages of anther development

Carbohydrate metabolism, signal transduction, folding, sorting and degradation, lipid metabolism, and biosynthesis of other secondary metabolites were the five most significant pathways (Fig. 7). In the a stage of anther development, the DEGs were mainly enriched in “pentose and glucuronate interconversions,” “phenylpropanoid biosynthesis,” “plant-pathogen interaction,” and “starch and sucrose metabolism” pathways. In the c stage, “protein processing in endoplasmic reticulum,” “phenylpropanoid biosynthesis,” and “pentose and glucuronate interconversions” were significantly enriched. When the anthers grew to the e stage, “starch and sucrose metabolism” and “pentose and glucuronate interconversions” were the two-most enriched pathways. In addition, “phenylpropanoid biosynthesis” was commonly enriched in all three development stages. “Cutin, suberine and wax biosynthesis” was significantly enriched in the a and e stages. These pathways were associated with cell wall and tapetum metabolism. These results suggested that anther development is associated with an extremely intricate and complex transcriptional network.

### Anther- and pollen development-related genes

Pollen development plays an important role in the reproduction of plants. It involves many events and is a highly complex process. In *Arabidopsis,* a number of genes and transcription factors regulating anther development have been reported. In this experiment, we identified a total of 260 DEGs related to anther and pollen development in the three comparison pairs (MS_a-vs-MF_a; MS_c-vs-MF_c; MS_e-vs-MF_e), including GDSL lipase genes, cytochrome-related genes, BHLH transcription factor family, MADS-box protein genes, and pectinesterase genes (Additional file 5: Table S1). There were 32 genes differentially expressed in the three stages of MS7–2, of which 29 DEGs were down-regulated, and three DEGs were up-regulated in MS7–2 **(**Table 2**)**. These results indicated that these DEGs mainly participated in the formation and differentiation of the tapetum, pollen tube growth, pollen development, and reproductive development in MF and vice versa in MS.

**Table 2:** Identification of DEGs associated with anther and pollen development in Wucai

Gene ID	Brassica Gene	Up/Down	Gene Name	Description
LOC103829123	BraA07g012490.3C	Down	*BCP1*	anther-specific protein BCP1-like
LOC103835426	BraA08g025170.3C	Down	*BCP1*	anther-specific protein BCP1-like
LOC103842268	BraA04g017810.3C	Down	*BHLH66*	transcription factor bHLH66-like
LOC103852197	BraA01g006790.3C	Down	*BHLH69*	transcription factor bHLH69
LOC103844110	BraA10g004440.3C	Down	*CHX23*	cation/H(+) antiporter 23, chloroplastic
LOC103852992	BraA02g023130.3C	Down	*EXL4*	GDSL esterase/lipase EXL4-like
LOC103862935	BraA02g023130.3C	Down	*EXL4*	GDSL esterase/lipase EXL4-like
LOC103849988	BraA05g039130.3C	Down	*GATL4*	probable galacturonosyltransferase-like 4
LOC103847046	BraA10g029710.3C	Down	*GRP17*	oleosin-B2-like
LOC103847047	BraA10g029720.3C	Down	*GRP17*	transcriptional regulatory protein AlgP-like
LOC103855247	BraA02g044650.3C	Down	*MRS2–2*	magnesium transporter MRS2–2-like
LOC103837566	BraA01g033950.3C	Down	*PEX1*	pollen-specific leucine-rich repeat extensin-like protein 1
LOC103860196	BraA03g038640.3C	Down	*PEX1*	pollen-specific leucine-rich repeat extensin-like protein 1
LOC103843098	BraA01g004250.3C	Down	*PEX4*	pollen-specific leucine-rich repeat extensin-like protein 4
LOC103834448	BraA08g016310.3C	Down	*PEX4*	pollen-specific leucine-rich repeat extensin-like protein 4
LOC103862310	BraA03g058490.3C	Down	*PEX4*	pollen-specific leucine-rich repeat extensin-like protein 4
LOC103858231	BraA03g024320.3C	Down	*PME4*	pectinesterase 5
LOC103866408	BraA05g000810.3C	Down	*PME4*	pectinesterase 5-like
LOC103833049	BraA08g004800.3C	Down	*PMEI1*	pectinesterase inhibitor 1
LOC103840768	BraA09g039750.3C	Down	*PMEI1*	pectinesterase inhibitor 1-like
LOC103847061	BraA10g029820.3C	Down	*PPME1*	pectinesterase PPME1
LOC103863930	BraA04g012380.3C	Down	*QRT2*	polygalacturonase QRT2
LOC103867511	BraA05g011180.3C	Down	*RALFL19*	protein RALF-like 19
LOC103835336	BraA08g024410.3C	Down	*RALFL4*	protein RALF-like 4
LOC103861226	BraA03g049500.3C	Down	*SBT3.1*	subtilisin-like protease SBT3.1
LOC103837935	BraA09g011690.3C	Down	*SHT*	spermidine hydroxycinnamoyl transferase-like
LOC103874054	BraA06g029650.3C	Down	*SHT*	spermidine hydroxycinnamoyl transferase-like
LOC103837770	BraA09g010490.3C	Down	*STP7*	sugar transport protein 7-like
LOC103862797	BraA04g000830.3C	Down	*VGDH2*	probable pectinesterase/pectinesterase inhibitor VGDH2
LOC103873153	BraA06g018600.3C	Up	*BT2*	BTB/POZ and TAZ domain-containing protein 2
LOC103844692	BraA10g000080.3C	Up	*SPL8*	squamosa promoter-binding-like protein 8
LOC103852769	BraA02g020890.3C	Up	*WSCP*	cysteine protease inhibitor WSCP-like

### Carbohydrate metabolism pathway-related genes

**Fig. 8:**
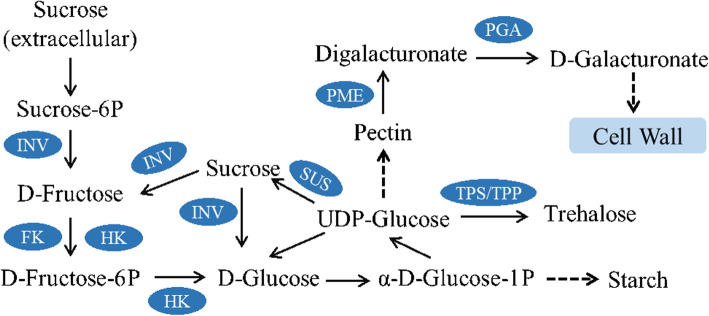
Related metabolic pathways and regulatory carbohydrate metabolism pathway of MS7–2 and MF7–2

According to the KEGG analyses, many DEGs were mainly enriched in the carbohydrate metabolism pathway. Carbohydrates provide a material basis for anther and pollen development and are also an important component of the cell wall. The regulatory pattern of related differential genes in the metabolic pathway is shown in Fig. 8.

The transcription levels of key enzymes in the carbohydrate metabolism pathway in MS7–2 and MF7–2 are shown in Additional file 2:Fig. S2. Five of the seven *INV* genes encoding beta-fructofuranosidase were up-regulated in MF and down-regulated in MS, and the *FK* and *HK* genes encoding fructokinase and hexokinase were all up-regulated in MF and down-regulated in MS. The expression patterns of *SUS* and *TPS* were down-regulated in MF, but up-regulated in MS. *TPP* in the trehalose biosynthesis pathway was down-regulated in MS. In addition, it should be noted that *PGA* and *PME*, two key enzymes in pectin metabolism, were all down-regulated in MS.

### Phenylpropanoid biosynthesis pathway-related genes

**Fig. 9:**
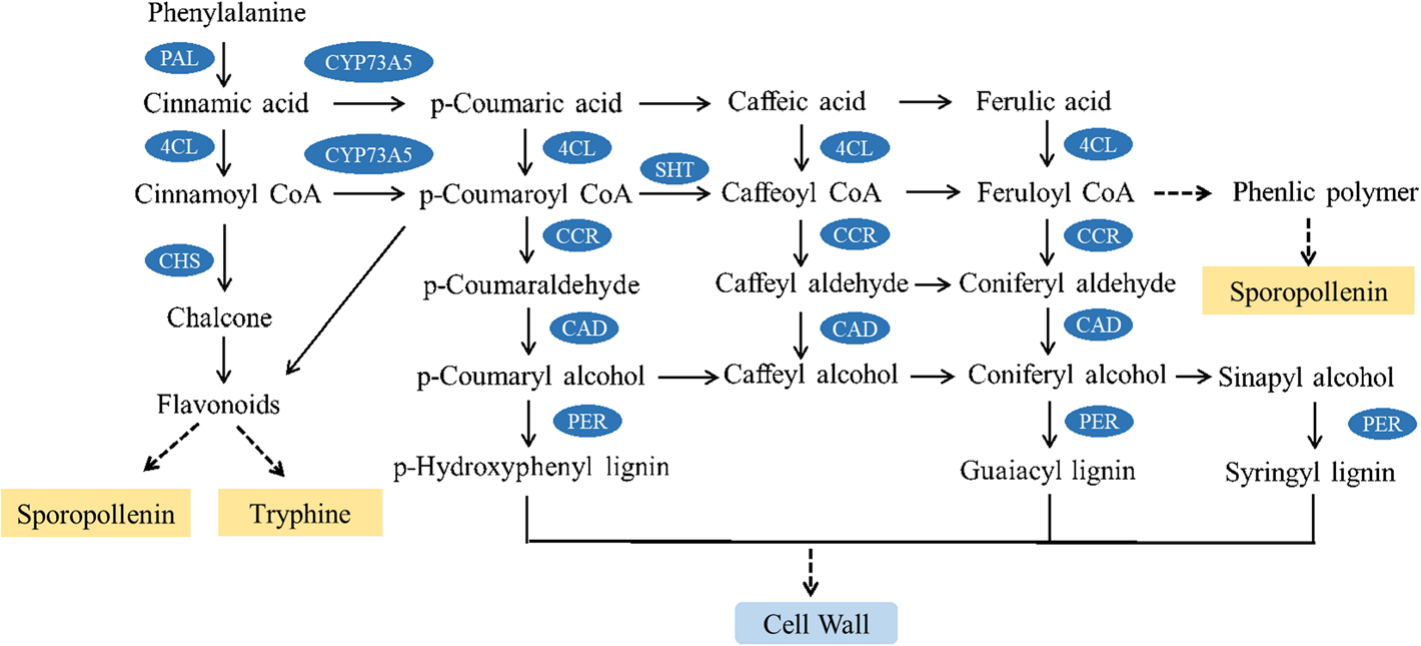
Related metabolic pathways and regulation of the phenylpropanoid biosynthesis pathway of MS7–2 and MF7–2

A large number of DEGs were also enriched in the phenylpropanoid biosynthesis pathway **(**Fig. 9**)**. Phenylpropanoid metabolism is catalyzed by a series of enzyme complexes. The interruption of these reactions may lead to the inhibition of the synthesis of some secondary metabolites in plants. Therefore, we carefully studied the genes involved in the regulation of this metabolic pathway. Our results identified 27 DEGs encoding phenylpropane metabolism-related enzymes.

In the phenylpropanoid biosynthesis pathway, nearly two-thirds of the genes were down-regulated in MS, including *PAL*, *CAD*, *SHT,* and *CHS* (Additional file 3: Fig. S3). A total of 14 *PER* genes were detected, half of which were down-regulated in MS and half of which were down-regulated in MF. In addition, spermidine hydroxycinnamoyl transferase, which can catalyze p-coumaroyl-CoA to produce caffeoyl-CoA, and *SHT*, which encodes this enzyme, were both down-regulated in MS.

### Oxidative phosphorylation pathway-related genes

**Fig. 10:**
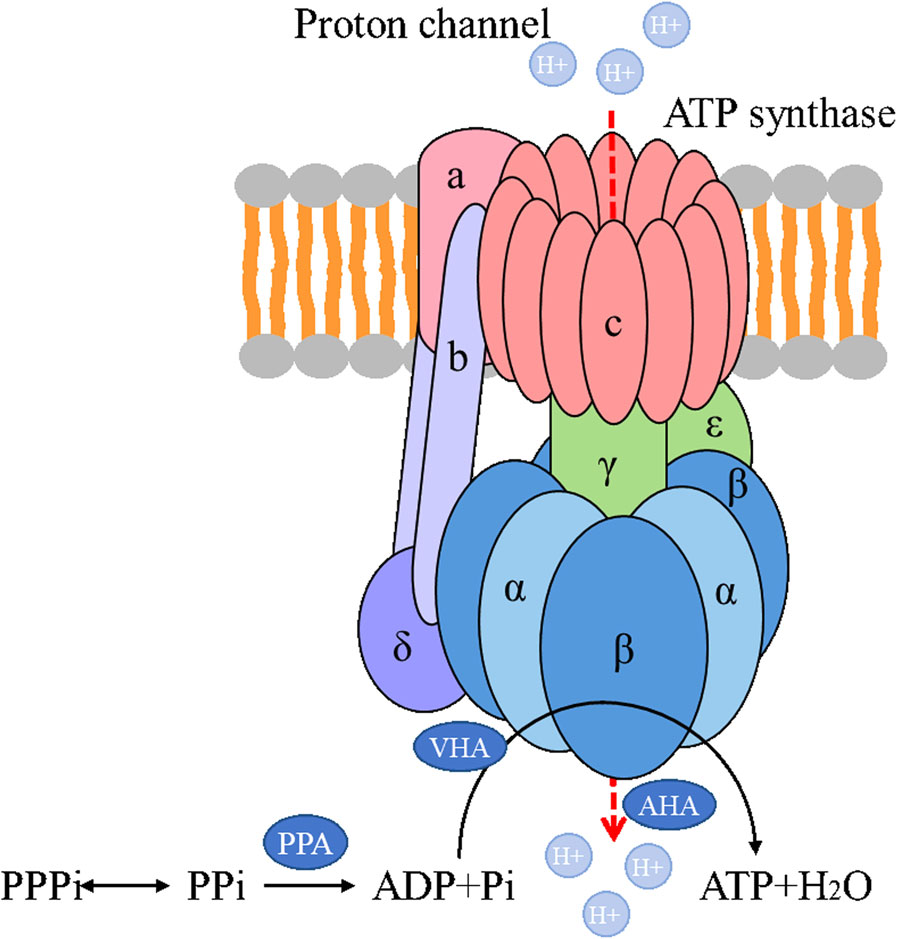
Related metabolic pathways and regulation oxidative phosphorylation pathway of MS7–2 and MF7–2

Energy metabolism disorder is also an important factor affecting pollen fertility. The energy produced by oxidative phosphorylation provides essential energy for plant growth and development. We plotted a model of oxidative phosphorylation metabolism **(**Fig. 10**)**. The DEGs were mainly enriched in ATP synthase (complex V). In the model, diphosphate is metabolized by soluble inorganic pyrophosphate (*PPA*) to produce ADP and Pi. H^+^ enter the membrane under the action of ATPase (*AHA*) along the proton channel. With the participation of V-type proton ATPase (*VHA*), ADP and PI produce ATP. All the DEGs in the oxidative phosphorylation pathway were concentrated in complex V and were all down-regulated in MS.

### Verification of DEGs by qRT-PCR

**Fig. 11:**
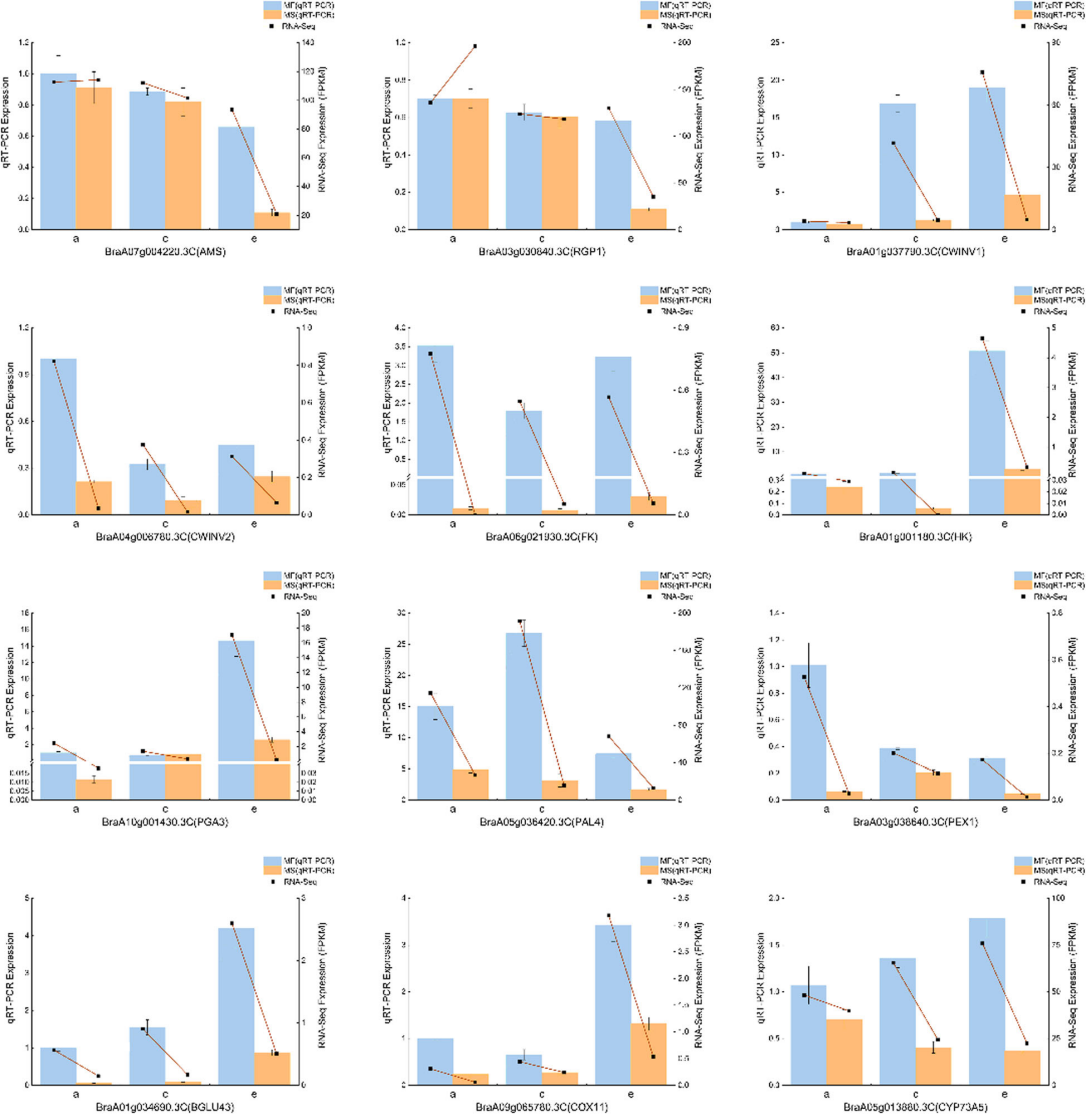
Expression levels of DEGs according to qRT-PCR (histogram) and RNA-Seq (red line chart). The FPKM values are based on the RNA-Seq data. The data obtained by the qRT-PCR represent the means based on three replicates

We selected 12 DEGs from the important metabolic pathways for qRT-PCR to verify the reliability of the RNA-Seq, including those encoding *AMS* transcription factor (BraA07g004220.3C, *AMS*), UDP-arabinopyranose mutase 1 (BraA03g030840.3C, *RGP1*), beta-fructofuranosidase insoluble isoenzyme (BraA01g037790.3C, *CWINV1* and BraA04g006780.3C, *CWINV2*), fructokinase (BraA06g021930.3C, *FK*), hexokinase (BraA01g001180.3C, *HK*), exopolygalacturonase 3 (BraA10g001430.3C, *PGA3*), phenylalanine ammonialyase 4 (BraA05g036420.3C, *PAL4*), pollen-specific leucine-rich repeat extensin-like protein 1 (BraA03g038640.3C, *PEX1*), beta-glucosidase 43 (BraA01g034690.3C, *BGLU43*), cytochrome c oxidase assembly protein 11 (BraA09g065780.3C, *COX11*), and trans-cinnamate 4-monooxygenase (BraA05g013880.3C, *CYP73A5*) **(**Fig. 11**)**. The gene expression patterns obtained from the qRT-PCR and RNA-Seq data showed similar trends, which confirmed the accuracy of the RNA sequencing results obtained in this study.

## Discussion

### Male sterility is caused by the disorder of carbohydrate metabolism

In plants, carbohydrate metabolism is one of the basic metabolic pathways in plant growth and development [39]. It plays an important role in the following aspects: (1) it participates in plant growth and development as an energy source and intermediate metabolite; (2) it has signaling functions; and (3) it acts as a regulator of growth, development, and gene expression [40]. In the process of pollen maturation, sugars provide energy and nutrition for anther growth and development. In addition, carbohydrates are also one of the important components of the plant cell wall. Therefore, the disorder of sugar metabolism can seriously damage pollen development and cause male sterility. We analyzed the regulatory network of the carbohydrate metabolism pathway **(**Fig. 8**)**. Sucrose from the extracellular domain enters the cell as a substrate to produce sucrose 6-phosphate, which is converted into D-fructose by *INV*. D-fructose is then converted into fructose phosphate by hexokinase (*HK*) and fructokinase (*FK*), and then glucose is produced by fructokinase. After a series of enzyme catalysis reactions, UDP glucose is finally produced. UDP glucose plays an important role in carbohydrate metabolism, some of which is converted into glucose to complete a cycle; some of which is catalyzed by sucrose synthase (*SUS*); some is produced by trehalose phosphate phosphatase (*TPP*) and trehalose phosphate synthase (*TPS*); and the remainder is used to produce pectin after a series of enzyme-catalyzed reactions. Pectin is an important component of the cell wall. *PME* can hydrolyze pectin to form digalacturonate, which is then catalyzed by *PGA* to produce d-galacturonate (GALUA). GALUA is an integral part of the primary cell wall and is essential for maintaining cell wall development.

FK and HK are both pivotal enzymes in carbohydrate metabolism [41]. As an important kinase that catalyzes the key metabolic step of fructose phosphorylation, FK helps phosphorylate fructose to form fructose 6-phosphate (F6P) and functions in all stages of plant development [42]. FK is involved in pollen germination and is suggested to be associated with starch accumulation [43]. In addition, the activity of FK is higher in normal pollen [44]. Pectin metabolism plays an important role in pollen tube regulation, as the top wall of the pollen tube is composed of a single layer of pectin [45]. After pectin is secreted into the cell wall in the form of methylesterification, it is modified by pectin methylesterases (PMEs) to release acid pectin and methanol [46]. PMEs have demonstrated their involvement in the determination of the shape of the pollen tube and the rate of its elongation [47]. Exogalacturonidase (PGA) catalyzes the degradation of galacturonic acid, the main component of pectin in plant cell wall, into a single polygalacturic acid residue, which exists in tapetum, sporophyte and pollen, and is related to pollen tube growth [48, 49]. In the present study, in the carbohydrate metabolism pathway, six and nine DEGs that were significantly down-regulated in MS7–2 were annotated as FK and PME, respectively, thirteen DEGs annotated as *PGA* were detected in the metabolic process of digalacturonate catalyzed to produce d-galacturonate. These DEGs were also significantly down regulated in MS7–2 **(**Additional file 2: Fig. S2). Therefore, we suggest that the down-regulation of *FK* may affect the accumulation of starch, the down-regulation of *PME* may affect the development of cell wall, and the down-regulation of *PGA* may lead to abnormal development of tapetum in MS7–2. It should be noted that the contents of total soluble sugar, sucrose, and starch in MS7–2 were significantly lower than in MF7–2, indicating that the down-regulation of some genes in MS7–2 led to the disorder of sugar metabolism in the anthers, which may have further led to male sterility.

### Phenylpropanoid metabolism plays an important role in the development of the tapetum

Phenylpropane metabolism has two important metabolic branches: one is in flavonoid biosynthesis, and the other is in the biosynthesis of lignin. First, phenylalanine is used as the substrate to produce *trans-*cinnamic acid under the catalysis of phenylalanine ammonia lyase (*PAL*) and is then converted into cinnamoyl-CoA by 4-coumarate-CoA ligase (*4CL*). After a series of reactions, cinnamoyl-CoA produces chalcone under the action of chalcone synthase (*CHS*) and then generates flavonoids, finally forming sporopollenin and tryphine in the anther wall. The metabolic regulatory network of lignin metabolism is more complex. p-Coumaric acid and p-coumaroyl-CoA are produced by the action of *trans*-cinnamate 4-monooxygenase (*CYP73A5*) and 4-coumarate-CoA ligase (*4CL*). In addition, p-coumaroyl-CoA is also a precursor for the synthesis of flavonoids. p-Hydroxyphenyl lignin is synthesized from coumarin by a series of enzymes, such as cinnamoyl CoA reductase (*CCR*), cinnamyl alcohol dehydrogenase (*CAD*), and peroxidase (*PER*). Guaiacyl lignin and syringyl lignin are produced by caffeic acid and ferulic acid through a series of metabolic steps. The synthesis of various lignin monomers will directly affect the development of the cell wall. Additionally, in the metabolic pathway, part of feruloyl-CoA is also involved in the synthesis of sporopollenin.

In previous studies, some enzymes related to flavonoid biosynthesis during the metabolism of phenylpropane showed specific activities at different stages of microspore development, including phenylalanine ammonia lyase, p-coumarate: CoA ligase, chalcone-flavanone isomerase, and flavonol synthase, and these enzymes exhibited high activity particularly at the stage of tapetum development. The tapetum plays an important role in the supply of enzymes necessary for the biosynthesis of flavonoids in the anthers. In addition, flavonoid metabolism will affect the formation of sporopollenin and tryphine formation [50]. Sporopollenin is a type of biopolymer mainly composed of polyhydroxy aliphatic compounds and phenols [51, 52] that exists in the pollen and spores [53] and is the main component of the pollen exine skeleton [22]. Flavonoids have key roles in pollen development and are essential for pollen maturation and pollen tube growth in flowering plants [54]. In another pathway, cytochrome P450s (P450s) are methylated monooxygenases involved in a large number of biosynthetic pathways in secondary and primary metabolism [55]. Studies have shown that CYP73 is a P40 family specifically involved in pollen development. *CYP73A5*, as a gene in the cytochrome P450 family, mainly functions in the early stage of catalyzing the synthesis of phenylpropanoid and sporopollenin synthesis [56]. *SHT* has been shown to be involved in the O-methylation of spermidine conjugates and affect flower development. Spermidine conjugates are located in the outermost layer of pollen coat, and *SHT* inhibition may lead to pollen coat development defects [57]. In addition, *SHT* inhibition in MS7–2 may also affect the synthesis of lignin monomers, thus affecting the development of cell wall **(**Fig. 9**)**. As an important branch of phenylpropanoid synthesis pathway, flavonoid synthesis pathway also plays an important role in anther development. *CHS* is a key enzyme in flavonoid synthesis. Its inhibition leads to the lack of flavonoid compounds and the dysfunction of tapetum [58]. We found that two and three DEGs in MS7–2 were annotated as *SHT* and *CHS*, and all of them were significantly down regulated. We suggest that the down regulated expression of *SHT* and *CHS* in MS7–2 may lead to abnormal pollen coat structure and tapetum function. The role of *SHT* and *CHS* in nuclear male sterility of wucai deserves further study (Additional file 3: Fig. S3). In this study, a large number of DEGs were enriched in the phenylpropanoid metabolism pathway, and many of these genes were down-regulated in MS7–2, including *PAL*, *CYP73A5*, *SHT*, *CHS,* and other key genes. The down-regulation of these genes may lead to the inhibition of the synthesis of flavonoids and lignin, as well as the normal formation of sporopollenin and the pollen wall, which may lead to the abortion of MS7–2 pollen.

### An abnormal electron transport chain leads to ROS accumulation and ultimately male sterility

The mitochondrion is important for energy production in cells and is also the main source of ROS [59, 60]. The electron transport chain on the mitochondrial membrane is the main site of ROS production [61]. ROS is involved in coordinating various plant processes, such as plant growth and apoptosis [16]. In addition, ROS, as a signaling molecule, is released from the mitochondria and transferred to the nucleus, triggering the formation of an abnormal tapetum and affecting pollen development [62, 63]. In higher plants, F1F0-ATPase (Complex V) is an important component of the mitochondrion that is involved in the energy generation of oxidative phosphorylation terminal events. F1F0-ATPase can catalyze the hydrolysis and synthesis of ATP reversibly according to the electrochemical gradient formed by proton passing through [64]. Studies have shown that several mitochondrial DNA regions encoding F1F0-ATPase press are associated with male sterility [65–68]. In this study, all the DEGs related to the electron transport chain in F1F0-ATPase were down-regulated, such as PPA, AHA, and VHA. The accumulation of cytoplasmic PPI is toxic and can lead to serious growth defects and even cell death [69]. PPAs are the enzymes that mainly participate in the hydrolysis of PPI into two inorganic phosphates (PI), which keep plant cells at a low level of cytoplasmic PPI [70]. The pollen-specific plasma membrane H^+^-ATPase isoforms AHAs are essential for pollen tube growth and fertility [71]. The down-regulation of PPA and AHA in ms7–2 may delay the transformation rate of PPI, make PPI accumulate in cells, and affect subsequent ATP synthesis, leading to the dysfunction of F1F0-ATPase, resulting in a change in mitochondrial internal and external potential and the inhibition of the electron transfer rate. The excessive electrons generated during pollen development combine with molecular oxygen to form ROS. ROS accumulation in cells leads to the abnormal degradation of the tapetum. We suggest that the dysfunction of F1F0-ATPase in MS7–2 may be an important reason for abortion.

MDA is usually considered as an indicator of lipid peroxidation caused by ROS, and its content can reflect the ROS level of a plant [1]. In addition, biochemical analysis showed that the content of MDA in MS7–2 was significantly higher than that in MF7–2, and both the H_2_O_2_ and O_2_^.-^ contents showed the same trend, indicating that there was a high accumulation of ROS in MS7–2.

## Conclusions

In this study, we first determined the abortion stage of the male sterile mutant MS7–2 by phenotypic and cytological observation, following which we analyzed the transcriptome of the mutant MS7–2 and fertile plant MF7–2 at different stages of flower bud development. Cytological analysis showed that the abortion of MS7–2 occurred after the tetrad stage when the tapetum developed abnormally, which eventually led to pollen abortion. A total of 2865, 3847, and 4981 DEGs were identified in the flower buds of MS7–2 and MF7–2 at the three development stages. The GO analysis showed that the DEGs were mainly enriched in cell process, metabolic process, and single biological process in the biological process category. The DEGs were highly enriched in cell, cell part, and organelle in cell component, and in the molecular function category, the DEGs enriched in binding and catalytic activity were the most abundant. The KEGG pathway analysis showed that the DEGs were enriched in carbohydrate metabolism, secondary metabolite synthesis, and signal transduction pathways. Physiological analysis showed that the contents of total soluble sugar, sucrose, and starch in the buds of MS7–2 were significantly higher than those of MF7–2, while the contents of MDA, H_2_O_2_, and O_2_^.-^ production rate were significantly lower than those of MF7–2. We analyzed the three key metabolic pathways of carbohydrate metabolism, phenylpropanoid metabolism, and oxidative phosphorylation and found that many key genes in these pathways of MS7–2 were down-regulated compared with MF7–2. Therefore, we inferred that genes in the key metabolic pathways were down-regulated, which inhibited the synthesis of carbohydrates and some secondary metabolites. The accumulated ROS in MS7–2 led to the abnormal development of the tapetum and synthesis of the pollen wall, eventually resulting in the abortion of MS7–2.

## Methods

### Plant materials

A male fertile *B. campestris* cultivar (wild-type, “MF7–2”) and a male sterile *B. campestris* cultivar (mutant, “MS7–2”) were used in the present study. The seed materials were from the Vegetable Breeding Laboratory, Horticulture College of Anhui Agricultural University. Fertile and sterile plants were cultivated in the same experimental plot at Anhui Agricultural University (Hefei, Anhui Province, China). During flowering, fertile and sterile flower buds of different sizes were chosen for paraffin embedding and sectioning. Young leaves of fertile and sterile plants were sampled for DNA extraction. Flower buds with lengths of less than 1.0 mm, 1.5 mm–2.0 mm, and 2.25–2.5 mm were selected from the fertile and sterile plants for transcriptome sequencing; these sizes represent the important periods for pollen sterility in the male sterile plants (Fig. 2 a1 a2, c1 c2, and e1 e2). The samples were then combined, wrapped in foil, snap-frozen in liquid nitrogen, and stored at 80 °C for future study. Each treatment had three biological replicates.

### Observations of floral organs and anther development

Whole plants were photographed using a camera (Nikon Digital Camera D3200, Nikon Corporation, Tokyo, Japan). At the full-bloom stage, the buds and floral organs of MS7–2 and MF7–2 (SZ650 continuous zoom stereomicroscope; Chongqing Optec Instrument Corporation, China) were observed by a stereomicroscope. For assessments of anther development, the flower buds of MF7–2 and MS7–2 were fixed using formalin-aceto-alcohol (FAA) solution, dyed using safranin and fast green, and observed under an Olympus optical microscope (Nikon ECLIPSE 80i; Nikon, Japan). Paraffin sections of these buds were obtained according to the methods of Li [26] and Zhang [30].

### Measurement of the contents of total soluble sugar, sucrose, starch, and malondialdehyde (MDA) and H_2_O_2_ and O_2_^.-^ production rate

The contents of total soluble sugar, sucrose, and starch were determined by anthrone colorimetry [72]. Fifty milligrams of dry bud sample was placed into a centrifuge tube, mixed with 4 mL of 80% ethanol, and placed in a water bath at 80 °C for 30 min. After cooling, the mixture was centrifuged at 5000×g for 3 min. The supernatant was collected, and the residue was washed with 80% ethanol three times. After each washing, the supernatants were extracted and combined, to which 10 mg of activated carbon was added, decolorized at 80 °C for 30 min, diluted to 10 mL, and then filtered to obtain the extract.

#### Total soluble sugar content

Twenty microliters of the above extract was combined with 480 μL of water and 2.5 mL of anthrone and reacted in a water bath at 90 °C for 15 min, following which it was allowed to cool and develop for 10 min and then measured at a wavelength of 620 nm.

#### Sucrose content

The above extract (0.25 mL) was added to 0.25 mL of water and mixed with 50 μL of 2 mol/L NaOH. After 5 min in a water bath at 90 °C, the extract was quickly cooled on ice, to which anthrone solution was added (150 mg anthrone added to 100 dilute sulfuric acid [760 mL specific gravity], and 1.84 concentrated sulfuric acid was added to 300 mL of water) and placed in a water bath at 80 °C for 10 min. After cooling, the solution was allowed to stand for 10 min, following which the absorbance was measured at a wavelength of 620 nm.

#### Starch content

Ten milliliters of 52% perchloric acid was added to the above residue, following which the supernatant was collected by centrifugation at 4000×g for 3 min. Ten milligrams of activated carbon was added, and the solution was filtered in a water bath at 80 °C for 30 min. Two milliliters of the filtrate was obtained, and 5 mL of anthrone was added after cooling in a water bath at 80 °C for 10 min. The absorbance was then measured at a wavelength of 620 nm.

#### MDA content

According to the method previously described by Mohammadi et al. [73], the MDA content in all samples was determined using the lipid membrane oxidation method. Fresh flower buds (0.5 g) were ground with 10 mL trichloroacetic acid (10%) and centrifuged at 4000 rpm for 10 min. Two milliliters of thiobarbituric acid (0.6%) was added to 2.0 mL of supernatant, which was placed in a water bath at 100 °C for 15 min and then cooled to room temperature. Finally, the absorbance of each aliquot was measured at 450 (A450), 532 (a532), and 600 nm (a600).

#### H_2_O_2_ content and O_2_^.-^ production rate

The H_2_O_2_ content and O_2_^.-^ production rate were measured using Solarbio reagent kits (BC3595 and BC1295, Solarbio, Beijing, China).

### RNA extraction, library construction, and Illumina sequencing

According to the manufacturer’s protocol, total RNA was extracted from six samples of MS7–2 and MF7–2 plant buds, each with three biological replicates, using the mirVana miRNA Isolation kit (Ambion, TX, USA). RNA purity, quantification, and integrity were evaluated using a NanoDrop 2000 spectrophotometer (Thermo Scientific, USA) and Agilent 2100 bioanalyzer (Agilent Technologies, Santa Clara, CA, USA). The samples with an RNA Integrity Number (RIN) ≥ 7 were subjected to subsequent analysis. The cDNA libraries were constructed using the TruSeq Stranded mRNA LT Sample Prep Kit (Illumina, CA, USA) following the manufacturer’s instructions. These libraries were then sequenced on an Illumina sequencing platform (HiSeqTM 2500 or Illumina HiSeq X Ten) to generate 125 bp/150 bp paired-end reads. Transcriptome sequencing and analysis were performed by OE Biotech Co., Ltd. (Shanghai, China).

### Sequence assembly and annotation

Through the Illumina platform, a large number of sample double-end sequencing data were obtained. Given the influence of data error rate on the results, Trimmomatic [74] software was used to preprocess the original data and summarize the reads in the entire quality control process. Using Hisat2 [75], clean reads were aligned with the *B. rapa* reference genome to obtain the location information of a gene as well as the specific sequence characteristics of the sequenced samples. The fragments per kilobase of transcript per million fragments sequenced (FPKM) [76] value of each gene was calculated using Cufflinks [77], and the read counts of each gene were obtained by HTSeq-count [78].

### Identification of differentially expressed genes (DEGs) and functional analysis

Differential expression analysis was performed for MS_a, MS_c, MS_e, MF_a, MF_c, and MF_e using the DESeq (2012) R package [79], the difference multiple was calculated, and significance testing of the number of reads was carried out using Nb (negative binomial distribution test). In order to control the false discovery rate (FDR), the *P*-value of each gene was calculated using the FDR value calculation method. The FDR error control method was used to correct the *P*-value for multiple hypothesis testing. *P* < 0.05 and |log2(foldchange)| > 1 were set as the thresholds for significant differential expression.

Hierarchical cluster analysis of DEGs was performed to demonstrate the expression patterns of genes in different groups and samples. Based on the Wallenius non-central hyper-geometric distribution, the GOseq R package was used for Gene Ontology (GO) enrichment analysis of the DEGs [80], and KOBAS software was used for Kyoto Encyclopedia of Genes and Genomes (KEGG) pathway enrichment of DEGs [81].

### Validation of DEGs by quantitative real-time (qRT) PCR

The six samples of MS7–2 and MF7–2 at the a, c, and e stages were prepared for qRT-PCR. The experimental samples were consistent with the samples of the RNA-Seq. The specific primers were designed with PRIMER 6.0, and their sequences are listed in Additional file 6: Table S2. According to the manufacturer’s instructions of the SYBR® Premix Ex Taq™ II Kit (TaKaRa, Japan), the experiment was performed on the Bio-Rad CFX96™ Real-Time System (Bio-Rad, CA, USA). The data were normalized with the expression level of *actin* as the internal control [30]. Three biological replicates were performed for each sample, and three technical replicates were performed for each gene. The relative expression level was calculated as 2^-ΔΔCt^ [82].

### Statistical analysis

Data were expressed as the mean ± SD with three biological replicates. Differences were analyzed using SPSS 22.0 (SPSS Institute, Inc., USA), and means were compared using the Student’s *t* test at a significance level of 0.05. The related figures were drawn using Origin Pro v9.1 software (OriginLab Corp., MA, USA).

## Supplementary Information

**Additional file 1: Fig. S1.** Heatmap analysis of anther- and pollen development-related genes.

**Additional file 2: Fig. S2.** Heatmap analysis of carbohydrate metabolism-related genes.

**Additional file 3: Fig. S3.** Heatmap analysis of phenylpropanoid biosynthesis-related genes.

**Additional file 4: Fig. S4.** Heatmap analysis of oxidative phosphorylation-related genes.

**Additional file 5: Table S1.** Identification of all DEGs associated with anther and pollen development in Wucai.

**Additional file 6: Table S2.** Primers used in this study.

## Data Availability

The raw RNA-Seq data used in this study have been deposited in the Nation Center for Biotechnology Information (NCBI) Sequence Read Archive (SRA) database under the accession number SUB8933024 (https://www.ncbi.nlm.nih.gov/sra/ SUB8933024).

## Abbreviations

ROS: Reactive oxygen species
CMS: Cytoplasmic male sterility
GMS: Genetic male sterility
CGMS: Cytoplasmic-genetic male sterility
PCD: Programmed cell death
DEGs: Differentially expressed genes
GO: Gene Ontology
KEGG: Kyoto Encyclopedia of Genes and Genomes
MDA: Malondialdehyde
qRT-PCR: Quantitative real-time polymerase chain reaction
RNA-Seq: RNA sequencing technology
