# Supplementary material for: Morphological characteristics and transcriptome analysis at different anther development stages of the male sterile mutant MS7–2 in Wucai (Brassica campestris L.)
Source: BMC Genomics. 2021 Sep 11;22:654. doi: 10.1186/s12864-021-07985-5 (PMC8436512; doi:10.1186/s12864-021-07985-5)
Supplement: Supplementary file 2 — Additional file 2: Fig. S2. Heatmap analysis of carbohydrate metabolism-related genes. [file 12864_2021_7985_MOESM2_ESM.docx]

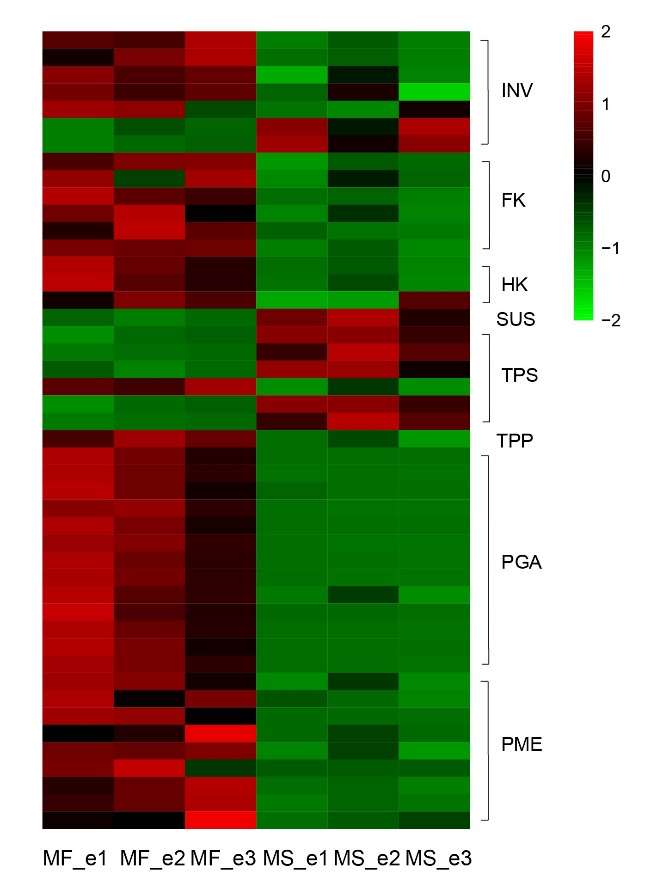


**Fig. S2** Heatmap analysis of carbohydrate metabolism-related genes. The expression levels shown are based on FPKM data. The color key represents the value of log2(FPKM). Red represents high expression, and green represents low expression. Each row represents a DEG.
