# Supplementary material for: Morphological characteristics and transcriptome analysis at different anther development stages of the male sterile mutant MS7–2 in Wucai (Brassica campestris L.)
Source: BMC Genomics. 2021 Sep 11;22:654. doi: 10.1186/s12864-021-07985-5 (PMC8436512; doi:10.1186/s12864-021-07985-5)
Supplement: Supplementary file 5 — Additional file 5: Table S1. Identification of all DEGs associated with anther and pollen development in Wucai. [file 12864_2021_7985_MOESM5_ESM.docx]

**Table S1** Identification of all DEGs associated with anther and pollen development in Wucai.

| Gene ID | Brassica Gene | Up/Down | Gene Name | Description |
| --- | --- | --- | --- | --- |
| LOC103836676 | BraA01g000060.3C | Down | CSLD4 | cellulose synthase-like protein D4 |
| LOC103874360 | BraA01g002820.3C | Down | ZAT3 | uncharacterized LOC103874360 |
| LOC103843098 | BraA01g004250.3C | Down | PEX4 | pollen-specific leucine-rich repeat extensin-like protein 4 |
| LOC103852197 | BraA01g006790.3C | Down | BHLH69 | transcription factor bHLH69 |
| LOC103855561 | BraA01g009230.3C | Down | CBL1 | calcineurin B-like protein 1 |
| LOC103857174 | BraA01g010570.3C | Down | APY7 | probable apyrase 7 |
| LOC103857931 | BraA01g011240.3C | Down | QRT3 | polygalacturonase QRT3 |
| LOC103866144 | BraA01g017130.3C | Down | WRKY34 | probable WRKY transcription factor 34 |
| LOC103866247 | BraA01g017220.3C | Down | NAS2 | nicotianamine synthase 1 |
| LOC103867975 | BraA01g019030.3C | Down | LLG3 | GPI-anchored protein LORELEI-like |
| LOC103868092 | BraA01g019140.3C | Down | ATA7 | uncharacterized LOC103868092 |
| LOC103836695 | BraA01g033180.3C | Down | PRK4 | pollen receptor-like kinase 4 |
| LOC103837566 | BraA01g033950.3C | Down | PEX1 | pollen-specific leucine-rich repeat extensin-like protein 1 |
| LOC103837607 | BraA01g033990.3C | Down | JGB | notchless protein homolog 1-like |
| LOC103838113 | BraA01g034470.3C | Down | VQ20 | VQ motif-containing protein 20 |
| LOC103839749 | BraA01g035360.3C | Down | PMEI2 | pectinesterase inhibitor 2-like |
| LOC103845300 | BraA01g038710.3C | Down | AGC1-5 | serine/threonine-protein kinase AGC1-5-like |
| LOC103845744 | BraA01g039120.3C | Down | RABA4D | ras-related protein RABA4d |
| LOC103848932 | BraA01g041190.3C | Down | PIP5K6 | phosphatidylinositol 4-phosphate 5-kinase 6 |
| LOC103849489 | BraA01g041630.3C | Down | PAP15 | purple acid phosphatase 15-like |
| LOC103849657 | BraA01g041790.3C | Down | PME23 | probable pectinesterase/pectinesterase inhibitor 23 |
| LOC103850654 | BraA02g002330.3C | Down | PPME1 | pectinesterase PPME1 |
| LOC108870746 | BraA02g002380.3C | Down | GRP17 | oleosin GRP-17-like |
| LOC103851267 | BraA02g008120.3C | Down | CPK17 | calcium-dependent protein kinase 34 |
| LOC103851650 | BraA02g011710.3C | Down | MSI1 | histone-binding protein MSI1-like |
| LOC103851685 | BraA02g012040.3C | Down | CER3 | protein ECERIFERUM 3 |
| LOC103852105 | BraA02g015420.3C | Down | ADF12 | actin-depolymerizing factor 12 |
| LOC103852758 | BraA02g020760.3C | Down | PATL1 | patellin-1-like |
| LOC103852992 | BraA02g023130.3C | Down | EXL4 | GDSL esterase/lipase EXL4-like |
| LOC103862935 | BraA02g023130.3C | Down | EXL4 | GDSL esterase/lipase EXL4-like |
| LOC103852994 | BraA02g023140.3C | Down | EXL6 | GDSL esterase/lipase EXL6 |
| LOC103853782 | BraA02g033630.3C | Down | CBL9 | calcineurin B-like protein 9 |
| LOC103854576 | BraA02g040010.3C | Down | GEX2 | protein GAMETE EXPRESSED 2 |
| LOC103854636 | BraA02g040490.3C | Down | NPF2.8 | protein NRT1/ PTR FAMILY 2.8-like |
| LOC103855247 | BraA02g044650.3C | Down | MRS2-2 | magnesium transporter MRS2-2-like |
| LOC103855703 | BraA03g002710.3C | Down | TGA10 | transcription factor TGA2.2-like |
| LOC103846326 | BraA03g007520.3C | Down | LIP1 | receptor-like kinase LIP1 |
| LOC103856200 | BraA03g007520.3C | Down | LIP1 | receptor-like kinase LIP1 |
| LOC103856202 | BraA03g007550.3C | Down | PIN5 | auxin efflux carrier component 5-like |
| LOC103857378 | BraA03g016740.3C | Down | CPK24 | calcium-dependent protein kinase 24 |
| LOC103857427 | BraA03g017190.3C | Down | MYB101 | myb-related protein 306 |
| LOC103858231 | BraA03g024320.3C | Down | PME4 | pectinesterase 5 |
| LOC103858686 | BraA03g028400.3C | Down | AGD13 | probable ADP-ribosylation factor GTPase-activating protein AGD13 |
| LOC103858781 | BraA03g029330.3C | Down | STP7 | sugar transport protein 7 |
| LOC103858948 | BraA03g030840.3C | Down | RGP1 | UDP-arabinopyranose mutase 1-like |
| LOC103858985 | BraA03g031150.3C | Down | LIP2 | receptor-like kinase LIP2 |
| LOC103859146 | BraA03g032610.3C | Down | GATL4 | probable galacturonosyltransferase-like 4 |
| LOC103859204 | BraA03g033140.3C | Down | CALS9 | callose synthase 9-like |
| LOC103859253 | BraA03g033540.3C | Down | PIP5K6 | phosphatidylinositol 4-phosphate 5-kinase 6 |
| LOC103859424 | BraA03g035100.3C | Down | SLSG | S-locus-specific glycoprotein |
| LOC103859432 | BraA03g035180.3C | Down | RABA4D | ras-related protein RABA4d-like |
| LOC103859438 | BraA03g035220.3C | Down | RBR1 | retinoblastoma-related protein 1-like |
| LOC103860196 | BraA03g038640.3C | Down | PEX1 | pollen-specific leucine-rich repeat extensin-like protein 1 |
| LOC103859893 | BraA03g039650.3C | Down | ACA9 | calcium-transporting ATPase 9, plasma membrane-type |
| LOC103860494 | BraA03g043400.3C | Down | AMS | transcription factor ABORTED MICROSPORES-like |
| LOC103860923 | BraA03g047600.3C | Down | CBL1 | calcineurin B-like protein 1 |
| LOC103861226 | BraA03g049500.3C | Down | SBT3.1 | subtilisin-like protease SBT3.1 |
| LOC103861494 | BraA03g051960.3C | Down | REN1 | rho GTPase-activating protein REN1-like |
| LOC103861628 | BraA03g053130.3C | Down | WRKY34 | probable WRKY transcription factor 34 |
| LOC103861687 | BraA03g053640.3C | Down | SPL | protein SPOROCYTELESS-like |
| LOC103861822 | BraA03g054320.3C | Down | LLG3 | GPI-anchored protein LORELEI-like |
| LOC103861826 | BraA03g054370.3C | Down | ATA7 | non-specific lipid-transfer protein 13 |
| LOC103862310 | BraA03g058490.3C | Down | PEX4 | pollen-specific leucine-rich repeat extensin-like protein 4 |
| LOC103862328 | BraA03g058690.3C | Down | PGDH1 | D-3-phosphoglycerate dehydrogenase 1, chloroplastic-like |
| LOC103862797 | BraA04g000830.3C | Down | VGDH2 | probable pectinesterase/pectinesterase inhibitor VGDH2 |
| LOC103862939 | BraA04g002330.3C | Down | SSL13 | protein STRICTOSIDINE SYNTHASE-LIKE 13 |
| LOC103863076 | BraA04g003670.3C | Down | PIP5K4 | phosphatidylinositol 4-phosphate 5-kinase 4-like |
| LOC103863839 | BraA04g011650.3C | Down | PTEN1 | phosphatidylinositol 3,4,5-trisphosphate 3-phosphatase and protein-tyrosine-phosphatase PTEN1 |
| LOC103863904 | BraA04g012030.3C | Down | DMP9 | uncharacterized LOC103863904 |
| LOC103863930 | BraA04g012380.3C | Down | QRT2 | polygalacturonase QRT2 |
| LOC103863968 | BraA04g012750.3C | Down | SWEET8 | bidirectional sugar transporter SWEET8 |
| LOC103864361 | BraA04g016500.3C | Down | KIP | protein KINKY POLLEN |
| LOC103842268 | BraA04g017810.3C | Down | BHLH66 | transcription factor bHLH66-like |
| LOC103864531 | BraA04g017810.3C | Down | BHLH66 | transcription factor bHLH66 |
| LOC103865113 | BraA04g022510.3C | Down | BHLH91 | transcription factor bHLH91-like |
| LOC103865114 | BraA04g022520.3C | Down | BHLH10 | transcription factor bHLH10 |
| LOC103865132 | BraA04g022700.3C | Down | CPK24 | calcium-dependent protein kinase 24-like |
| LOC103865169 | BraA04g023020.3C | Down | CHX21 | cation/H(+) antiporter 21-like |
| LOC103865270 | BraA04g024030.3C | Down | RIC1 | CRIB domain-containing protein RIC1-like |
| LOC103865435 | BraA04g025010.3C | Down | AGD10 | ADP-ribosylation factor GTPase-activating protein AGD10 |
| LOC103865564 | BraA04g026170.3C | Down | METK3 | S-adenosylmethionine synthase 3-like |
| LOC103865574 | BraA04g026260.3C | Down | PAL1 | phenylalanine ammonia-lyase 1 |
| LOC103866408 | BraA05g000810.3C | Down | PME4 | pectinesterase 5-like |
| LOC103867229 | BraA05g008320.3C | Down | PAL1 | phenylalanine ammonia-lyase 1 |
| LOC103867511 | BraA05g011180.3C | Down | RALFL19 | protein RALF-like 19 |
| LOC103867533 | BraA05g011390.3C | Down | RIC1 | CRIB domain-containing protein RIC1-like |
| LOC103867568 | BraA05g011700.3C | Down | CSLD1 | cellulose synthase-like protein D1 |
| LOC103867868 | BraA05g013140.3C | Down | CPK24 | calcium-dependent protein kinase 24-like |
| LOC103867954 | BraA05g013880.3C | Down | CYP73A5 | trans-cinnamate 4-monooxygenase |
| LOC103832753 | BraA05g016470.3C | Down | ALA6 | phospholipid-transporting ATPase 6-like |
| LOC103868697 | BraA05g020420.3C | Down | PMEI1 | pectinesterase inhibitor 1-like |
| LOC103868839 | BraA05g022170.3C | Down | DTM1 | signal peptidase complex-like protein DTM1 |
| LOC103835362 | BraA05g025950.3C | Down | ACA9 | calcium-transporting ATPase 9, plasma membrane-type |
| LOC103870236 | BraA05g034400.3C | Down | AGC1-5 | serine/threonine-protein kinase AGC1-5 |
| LOC103870267 | BraA05g034700.3C | Down | RABA4D | ras-related protein RABA4d-like |
| LOC103849988 | BraA05g039130.3C | Down | GATL4 | probable galacturonosyltransferase-like 4 |
| LOC103872132 | BraA06g010490.3C | Down | UTR3 | UDP-galactose/UDP-glucose transporter 3 |
| LOC103872400 | BraA06g012030.3C | Down | CNGC7 | putative cyclic nucleotide-gated ion channel 7 |
| LOC103873137 | BraA06g018410.3C | Down | CYP94B3 | cytochrome P450 94B3 |
| LOC103873190 | BraA06g019090.3C | Down | LBD27 | LOB domain-containing protein 27 |
| LOC103873243 | BraA06g019580.3C | Down | TIP5-1 | probable aquaporin TIP5-1 |
| LOC103873410 | BraA06g020840.3C | Down | MPK3 | mitogen-activated protein kinase 3 |
| LOC103873514 | BraA06g024750.3C | Down | LCB2b | long chain base biosynthesis protein 2b-like |
| LOC103873758 | BraA06g026960.3C | Down | SLSG | S-locus-specific glycoprotein S13-like |
| LOC103874054 | BraA06g029650.3C | Down | SHT | spermidine hydroxycinnamoyl transferase-like |
| LOC103874446 | BraA06g033110.3C | Down | SPH12 | uncharacterized LOC103874446 |
| LOC103874459 | BraA06g033250.3C | Down | STP8 | sugar transport protein 8 |
| LOC103874576 | BraA06g034360.3C | Down | NPF2.8 | protein NRT1/ PTR FAMILY 2.8-like |
| LOC103837242 | BraA06g035730.3C | Down | AAT1 | acetyl-CoA acetyltransferase, cytosolic 1-like |
| LOC103854526 | BraA06g035730.3C | Down | AAT1 | acetyl-CoA acetyltransferase, cytosolic 1-like |
| LOC103828115 | BraA06g044980.3C | Down | CSLD4 | cellulose synthase-like protein D4 |
| LOC103828177 | BraA07g000430.3C | Down | PHL4 | myb family transcription factor PHL4 |
| LOC103846021 | BraA07g004220.3C | Down | AMS | transcription factor ABORTED MICROSPORES |
| LOC103846054 | BraA07g004670.3C | Down | PLT1 | putative polyol transporter 1 |
| LOC103848531 | BraA07g006770.3C | Down | PRK2 | pollen receptor-like kinase 2 |
| LOC103829123 | BraA07g012490.3C | Down | BCP1 | anther-specific protein BCP1-like |
| LOC103830078 | BraA07g022610.3C | Down | PIP5K4 | phosphatidylinositol 4-phosphate 5-kinase 4 |
| LOC103830095 | BraA07g022820.3C | Down | AGL18 | agamous-like MADS-box protein AGL18 |
| LOC103831403 | BraA07g024270.3C | Down | DUO1 | transcription factor MYB57-like |
| LOC103830347 | BraA07g025040.3C | Down | VGDH2 | probable pectinesterase/pectinesterase inhibitor VGDH2 |
| LOC103830404 | BraA07g025500.3C | Down | ROPGEF12 | rop guanine nucleotide exchange factor 12 |
| LOC103830824 | BraA07g029350.3C | Down | SUC1 | sucrose transport protein SUC1-like |
| LOC103831048 | BraA07g031570.3C | Down | 4CL3 | 4-coumarate--CoA ligase 3 |
| LOC103831354 | BraA07g034450.3C | Down | AGL30 | agamous-like MADS-box protein AGL30 |
| LOC103831701 | BraA07g036350.3C | Down | SUC1 | sucrose transport protein SUC1-like |
| LOC103831734 | BraA07g036640.3C | Down | WSCP | cysteine protease inhibitor WSCP-like |
| LOC103832619 | BraA08g001020.3C | Down | ALA6 | phospholipid-transporting ATPase 6 |
| LOC103833049 | BraA08g004800.3C | Down | PMEI1 | pectinesterase inhibitor 1 |
| LOC103833836 | BraA08g010260.3C | Down | GLCNAC1PUT1 | UDP-N-acetylglucosamine diphosphorylase 1 |
| LOC103834448 | BraA08g016310.3C | Down | PEX4 | pollen-specific leucine-rich repeat extensin-like protein 4 |
| LOC103834716 | BraA08g018570.3C | Down | PLA2-GAMMA | phospholipase A2-gamma-like |
| LOC103834902 | BraA08g020380.3C | Down | REN1 | rho GTPase-activating protein REN1-like |
| LOC103829023 | BraA08g024410.3C | Down | RALFL4 | protein RALF-like 4 |
| LOC103835336 | BraA08g024410.3C | Down | RALFL4 | protein RALF-like 4 |
| LOC103838970 | BraA08g024410.3C | Down | RALFL4 | protein RALF-like 4 |
| LOC103835426 | BraA08g025170.3C | Down | BCP1 | anther-specific protein BCP1-like |
| LOC103829350 | BraA08g026930.3C | Down | AGL104 | agamous-like MADS-box protein AGL104 |
| LOC103832222 | BraA08g026930.3C | Down | AGL66 | agamous-like MADS-box protein AGL66 |
| LOC103835708 | BraA08g026930.3C | Down | AGL104 | agamous-like MADS-box protein AGL104 |
| LOC103836449 | BraA08g033660.3C | Down | STP2 | sugar transport protein 2 |
| LOC103836549 | BraA08g034620.3C | Down | HMGB15 | high mobility group B protein 15-like |
| LOC103836572 | BraA08g034860.3C | Down | RIC3 | CRIB domain-containing protein RIC3-like |
| LOC103837177 | BraA09g005040.3C | Down | MIRO1 | mitochondrial Rho GTPase 1-like |
| LOC103837770 | BraA09g010490.3C | Down | STP7 | sugar transport protein 7-like |
| LOC103837935 | BraA09g011690.3C | Down | SHT | spermidine hydroxycinnamoyl transferase-like |
| LOC103837973 | BraA09g011960.3C | Down | PLA2-BETA | phospholipase A2-beta-like |
| LOC103838057 | BraA09g012160.3C | Down | RTEL1 | Fanconi anemia group J protein homolog |
| LOC103838293 | BraA09g012980.3C | Down | UGE3 | bifunctional UDP-glucose 4-epimerase and UDP-xylose 4-epimerase 3 |
| LOC103838729 | BraA09g019060.3C | Down | XI-E | myosin-11 |
| LOC103839428 | BraA09g023720.3C | Down | AGL30 | agamous-like MADS-box protein AGL30 |
| LOC103839751 | BraA09g024880.3C | Down | AGD13 | probable ADP-ribosylation factor GTPase-activating protein AGD13 |
| LOC103840462 | BraA09g035260.3C | Down | SEI2 | seipin-2-like |
| LOC103835427 | BraA09g037400.3C | Down | JGB | myosin heavy chain kinase B-like |
| LOC103840768 | BraA09g039750.3C | Down | PMEI1 | pectinesterase inhibitor 1-like |
| LOC103841655 | BraA09g048090.3C | Down | ADPG1 | polygalacturonase ADPG1 |
| LOC103841896 | BraA09g050260.3C | Down | DUO1 | myb-related protein Myb4 |
| LOC103842447 | BraA09g054470.3C | Down | ACA7 | putative calcium-transporting ATPase 7, plasma membrane-type |
| LOC103843227 | BraA09g061220.3C | Down | SPH21 | pumilio homolog 15 |
| LOC103843594 | BraA09g064330.3C | Down | IP5P13 | type I inositol polyphosphate 5-phosphatase 13-like |
| LOC103843632 | BraA09g064710.3C | Down | HMGB15 | high mobility group B protein 15-like |
| LOC103844587 | BraA09g065780.3C | Down | COX11 | cytochrome c oxidase assembly protein COX11, mitochondrial |
| LOC103844638 | BraA09g066080.3C | Down | CYP703A2 | cytochrome P450 703A2 |
| LOC103844207 | BraA10g003410.3C | Down | HMGB15 | high mobility group B protein 15 |
| LOC103844110 | BraA10g004440.3C | Down | CHX23 | cation/H(+) antiporter 23, chloroplastic |
| LOC103844066 | BraA10g004880.3C | Down | BHLH89 | transcription factor bHLH89 |
| LOC103843953 | BraA10g006040.3C | Down | STP2 | sugar transport protein 2 |
| LOC103845220 | BraA10g015900.3C | Down | CER3 | protein ECERIFERUM 3-like |
| LOC103845738 | BraA10g020750.3C | Down | GNL2 | ARF guanine-nucleotide exchange factor GNL2 |
| LOC103846442 | BraA10g024250.3C | Down | PIN8 | auxin efflux carrier component 8-like |
| LOC103846453 | BraA10g024360.3C | Down | CNGC18 | cyclic nucleotide-gated ion channel 18 |
| LOC103846881 | BraA10g028210.3C | Down | ATL73 | RING-H2 finger protein ATL73 |
| LOC103846926 | BraA10g028600.3C | Down | PIRL1 | plant intracellular Ras-group-related LRR protein 1 |
| LOC103847025 | BraA10g029530.3C | Down | TGA10 | transcription factor TGA2.2 |
| LOC103847046 | BraA10g029710.3C | Down | GRP17 | oleosin-B2-like |
| LOC103847047 | BraA10g029720.3C | Down | GRP17 | transcriptional regulatory protein AlgP-like |
| LOC103847061 | BraA10g029820.3C | Down | PPME1 | pectinesterase PPME1 |
| LOC103847416 | BraA10g032920.3C | Down | CER26L | uncharacterized LOC103847416 |
| LOC103847494 | BraA10g033660.3C | Down | NPF8.2 | protein NRT1/ PTR FAMILY 8.2 |
| LOC103828251 | BraA01g002980.3C | Up | TKPR1 | tetraketide alpha-pyrone reductase 1 |
| LOC103833867 | BraA01g003480.3C | Up | PKSB | type III polyketide synthase B-like |
| LOC103853011 | BraA01g007630.3C | Up | UND | aspartyl protease UND |
| LOC103867043 | BraA01g018070.3C | Up | SPL | protein SPOROCYTELESS |
| LOC103829793 | BraA01g028200.3C | Up | At1g60420 | probable nucleoredoxin 1 |
| LOC103831105 | BraA01g029560.3C | Up | OST3B | probable dolichyl-diphosphooligosaccharide--protein glycosyltransferase subunit 3B |
| LOC103832133 | BraA01g030220.3C | Up | A6 | probable glucan endo-1,3-beta-glucosidase A6 |
| LOC103845973 | BraA01g039610.3C | Up | MYB65 | transcription factor GAMYB-like |
| LOC103847142 | BraA01g039830.3C | Up | DREB2B | dehydration-responsive element-binding protein 2B-like |
| LOC103850594 | BraA02g001830.3C | Up | CYP90A1 | cytochrome P450 90A1-like |
| LOC103851523 | BraA02g010460.3C | Up | LIP2 | putative serine/threonine-protein kinase |
| LOC103852027 | BraA02g014540.3C | Up | OPT8 | oligopeptide transporter 8 |
| LOC103852312 | BraA02g016940.3C | Up | MMD1 | PHD finger protein MALE MEIOCYTE DEATH 1 |
| LOC103852769 | BraA02g020890.3C | Up | WSCP | cysteine protease inhibitor WSCP-like |
| LOC103249164 | BraA02g020900.3C | Up | WSCP | trypsin inhibitor B |
| LOC103852904 | BraA02g022310.3C | Up | MYB80 | transcription factor MYB34-like |
| LOC103839540 | BraA02g033720.3C | Up | RABD2B | ras-related protein RABD2b |
| LOC103856329 | BraA03g008750.3C | Up | RUK | serine/threonine-protein kinase RUNKEL-like |
| LOC103858015 | BraA03g022270.3C | Up | BZIP34 | basic leucine zipper 34-like |
| LOC103858037 | BraA03g022490.3C | Up | AHL16 | AT-hook motif nuclear-localized protein 16-like |
| LOC103858440 | BraA03g026210.3C | Up | CEP1 | KDEL-tailed cysteine endopeptidase CEP1 |
| LOC103858882 | BraA03g030210.3C | Up | GRXC7 | glutaredoxin-C7 |
| LOC103859255 | BraA03g033550.3C | Up | QRT2 | polygalacturonase QRT2-like |
| LOC103859373 | BraA03g034600.3C | Up | DREB2B | dehydration-responsive element-binding protein 2B-like |
| LOC103859880 | BraA03g039540.3C | Up | NFYA9 | nuclear transcription factor Y subunit A-9-like |
| LOC103860005 | BraA03g040850.3C | Up | A6 | probable glucan endo-1,3-beta-glucosidase A6 |
| LOC103860437 | BraA03g042950.3C | Up | MEE14 | CCG-binding protein 1-like |
| LOC103861314 | BraA03g050260.3C | Up | INP1 | protein INAPERTURATE POLLEN1-like |
| LOC103861316 | BraA03g050280.3C | Up | INP1 | protein INAPERTURATE POLLEN1-like |
| LOC103861867 | BraA03g054860.3C | Up | SPH1 | uncharacterized LOC103861867 |
| LOC103862347 | BraA03g058820.3C | Up | SUC1 | sucrose transport protein SUC1-like |
| LOC103862380 | BraA03g059080.3C | Up | PKSB | type III polyketide synthase B-like |
| LOC103863288 | BraA04g000790.3C | Up | At3g62230 | F-box protein At3g62230 |
| LOC103863501 | BraA04g007290.3C | Up | JGB | uncharacterized WD repeat-containing protein all2124-like |
| LOC103863565 | BraA04g008040.3C | Up | A6 | probable glucan endo-1,3-beta-glucosidase A6 |
| LOC103865637 | BraA04g026900.3C | Up | PKSA | type III polyketide synthase A |
| LOC103866262 | BraA04g031940.3C | Up | ARID1 | AT-rich interactive domain-containing protein 1-like |
| LOC103867010 | BraA05g006260.3C | Up | P5CSA | delta-1-pyrroline-5-carboxylate synthase A |
| LOC103867413 | BraA05g010210.3C | Up | At2g34850 | putative UDP-arabinose 4-epimerase 2 |
| LOC103865042 | BraA05g013880.3C | Up | CYP73A5 | trans-cinnamate 4-monooxygenase-like |
| LOC103868543 | BraA05g018070.3C | Up | IOS1 | probable LRR receptor-like serine/threonine-protein kinase At1g51860 |
| LOC103869111 | BraA05g025100.3C | Up | POP2 | gamma-aminobutyrate transaminase POP2, mitochondrial |
| LOC103870411 | BraA05g035790.3C | Up | DREB2B | dehydration-responsive element-binding protein 2B |
| LOC103870162 | BraA05g037320.3C | Up | ERDJ3A | dnaJ protein ERDJ3A-like |
| LOC103870573 | BraA05g037320.3C | Up | ERDJ3A | dnaJ protein ERDJ3A |
| LOC103870621 | BraA05g037790.3C | Up | QRT2 | polygalacturonase QRT2-like |
| LOC103871723 | BraA06g006390.3C | Up | TKPR1 | tetraketide alpha-pyrone reductase 1-like |
| LOC103842973 | BraA06g010490.3C | Up | UTR3 | UDP-galactose/UDP-glucose transporter 3-like |
| LOC103872324 | BraA06g011390.3C | Up | PV42A | SNF1-related protein kinase regulatory subunit gamma-like PV42a |
| LOC103873153 | BraA06g018600.3C | Up | BT2 | BTB/POZ and TAZ domain-containing protein 2 |
| LOC103873737 | BraA06g026770.3C | Up | BT1 | BTB/POZ and TAZ domain-containing protein 1 |
| LOC103828289 | BraA07g001150.3C | Up | PLA2-BETA | phospholipase A2-beta |
| LOC103828454 | BraA07g002990.3C | Up | WUS | protein WUSCHEL-like |
| LOC103828708 | BraA07g009320.3C | Up | A6 | probable glucan endo-1,3-beta-glucosidase A6 |
| LOC103829490 | BraA07g016380.3C | Up | At2g21870 | uncharacterized LOC103829490 |
| LOC103832001 | BraA07g027720.3C | Up | SRS5 | protein SHI RELATED SEQUENCE 5 |
| LOC103831214 | BraA07g033210.3C | Up | POD1 | protein POLLEN DEFECTIVE IN GUIDANCE 1 |
| LOC103831736 | BraA07g036660.3C | Up | WSCP | cysteine protease inhibitor WSCP |
| LOC103831738 | BraA07g036670.3C | Up | WSCP | cysteine protease inhibitor WSCP-like |
| LOC103831739 | BraA07g036670.3C | Up | WSCP | cysteine protease inhibitor WSCP-like |
| LOC103833780 | BraA08g009700.3C | Up | A6 | probable glucan endo-1,3-beta-glucosidase A6 |
| LOC103834386 | BraA08g015650.3C | Up | PKSB | type III polyketide synthase B |
| LOC103835616 | BraA08g025960.3C | Up | ORC6 | origin of replication complex subunit 6 |
| LOC103836518 | BraA08g034250.3C | Up | BT3 | BTB/POZ and TAZ domain-containing protein 3-like |
| LOC103837285 | BraA09g004520.3C | Up | BZIP1 | basic leucine zipper 1-like |
| LOC103837180 | BraA09g005080.3C | Up | MIRO1 | mitochondrial Rho GTPase 1-like |
| LOC103837634 | BraA09g010050.3C | Up | CALS5 | callose synthase 5 |
| LOC103837833 | BraA09g011150.3C | Up | WUS | protein WUSCHEL-like |
| LOC103838045 | BraA09g012710.3C | Up | 4CLL1 | 4-coumarate--CoA ligase-like 1 |
| LOC103839426 | BraA09g023740.3C | Up | SPP | signal peptide peptidase-like |
| LOC103848134 | BraA09g036210.3C | Up | WSCP | cysteine protease inhibitor WSCP-like |
| LOC103842660 | BraA09g056830.3C | Up | TIFY10A | protein TIFY 10A-like |
| LOC103842751 | BraA09g057750.3C | Up | LOX3 | lipoxygenase 3, chloroplastic |
| LOC103844692 | BraA10g000080.3C | Up | SPL8 | squamosa promoter-binding-like protein 8 |
| LOC103844101 | BraA10g004520.3C | Up | BT3 | BTB/POZ and TAZ domain-containing protein 3-like |
| LOC103844032 | BraA10g005240.3C | Up | GPAT1 | glycerol-3-phosphate acyltransferase 1 |
| LOC103848657 | BraA10g009060.3C | Up | SWEET13 | bidirectional sugar transporter SWEET13 |
| LOC103844949 | BraA10g013050.3C | Up | MYB120 | transcription factor MYB26-like |
| LOC103844985 | BraA10g013410.3C | Up | GEX1 | protein GAMETE EXPRESSED 1 |
| LOC103845560 | BraA10g019050.3C | Up | MS1 | PHD finger protein MALE STERILITY 1 |
| LOC103846565 | BraA10g025310.3C | Up | TKPR2 | tetraketide alpha-pyrone reductase 2 |
| LOC103846910 | BraA10g028480.3C | Up | CYP90A1 | cytochrome P450 90A1 |
