# Supplementary material for: Morphological characteristics and transcriptome analysis at different anther development stages of the male sterile mutant MS7–2 in Wucai (Brassica campestris L.)
Source: BMC Genomics. 2021 Sep 11;22:654. doi: 10.1186/s12864-021-07985-5 (PMC8436512; doi:10.1186/s12864-021-07985-5)
Supplement: Supplementary file 6 — Additional file 6: Table S2. Primers used in this study. [file 12864_2021_7985_MOESM6_ESM.docx]

**Table S2** Primers used in this study.

| Gene name | Primer name | Primer sequences (5’-3’) |
| --- | --- | --- |
| *BnaActin* | F | TGGGTTTGCTGGTGACGAT |
|  | R | TGCCTAGGACGACCAACAATACT |
| BraA07g004220.3C(*AMS*) | F | CAGGTCGCTTGTTCCCA |
|  | R | CTTGCTGCCTATTAGATCCAT |
| BraA03g030840.3C(*RGP1*) | F | CCTTACCATCTCATCATCGTCCA |
|  | R | AGCAACAAAGCAATCGTCGTC |
| BraA01g037790.3C(*CWINV1*) | F | TCATCGGAAGCAAGATCCAC |
|  | R | GTTTCGTGTCATCCAAGCTA |
| BraA04g006780.3C(*CWINV2*) | F | TTTCCGACCCATACCTCA |
|  | R | GCAATTCCTCTACGCTT |
| BraA06g021930.3C(*FK*) | F | ACTTCGTTCCCACCGTCT |
|  | R | TTTCAAGATTCCGGCGAGCA |
| BraA01g001180.3C(*HK*) | F | TGTGTTTTCTCAGCCTTACGTT |
|  | R | TTCAAGATCCCCGCTATTCCC |
| BraA10g001430.3C(*PGA3*) | F | CCGCTAAGGCAAATAACACT |
|  | R | TCCCACTGCTCCAAACTGA |
| BraA05g036420.3C(*PAL4*) | F | CAATTCAAAAGCCGTGGGTC |
|  | R | CTGTCCCGTTCACAAGAGC |
| BraA03g038640.3C(*PEX1*) | F | ATCCCTGAGACAATCGGTA |
|  | R | TCGCATCAAACACAGTCACA |
| BraA01g034690.3C(*BGLU43*) | F | TCAGCCGTCCAGAGATACCG |
|  | R | AAGCCTCTCCTTCACGATG |
| BraA09g065780.3C(*COX11*) | F | TATTGCTCCATGCTCTCGTT |
|  | R | CGCGTAAGTTAGCCCCA |
| BraA05g013880.3C(*CYP73A5*) | F | ACGGTTCCTTTCTTCACCAAC |
|  | R | AACCTGCTCCTCTCTCCGTTC |
